# Multi-objective optimization framework to obtain model-based guidelines for tuning biological synthetic devices: an adaptive network case

**DOI:** 10.1186/s12918-016-0269-0

**Published:** 2016-03-11

**Authors:** Yadira Boada, Gilberto Reynoso-Meza, Jesús Picó, Alejandro Vignoni

**Affiliations:** Institut d’Automàtica i Informàtica Industrial, Universitat Politècnica de València, Valencia, Spain; Industrial and Systems Engineering Graduate Program (PPGEPS), Pontificial Catholic University of Parana (PUCPR), Curitiba, Brazil; Present Address: Center for Systems Biology Dresden (CSBD), Max Planck Institute of Molecular Cell Biology and Genetics, Dresden, Germany

**Keywords:** Biological circuits, Dynamic behavior, Multi-objective optimization, Kinetic parameters, Biological tuning knobs

## Abstract

**Background:**

Model based design plays a fundamental role in synthetic biology. Exploiting modularity, i.e. using biological parts and interconnecting them to build new and more complex biological circuits is one of the key issues. In this context, mathematical models have been used to generate predictions of the behavior of the designed device. Designers not only want the ability to predict the circuit behavior once all its components have been determined, but also to help on the design and selection of its biological parts, i.e. to provide guidelines for the experimental implementation. This is tantamount to obtaining proper values of the model parameters, for the circuit behavior results from the interplay between model structure and parameters tuning. However, determining crisp values for parameters of the involved parts is not a realistic approach. Uncertainty is ubiquitous to biology, and the characterization of biological parts is not exempt from it. Moreover, the desired dynamical behavior for the designed circuit usually results from a trade-off among several goals to be optimized.

**Results:**

We propose the use of a multi-objective optimization tuning framework to get a model-based set of guidelines for the selection of the kinetic parameters required to build a biological device with desired behavior. The design criteria are encoded in the formulation of the objectives and optimization problem itself. As a result, on the one hand the designer obtains qualitative regions/intervals of values of the circuit parameters giving rise to the predefined circuit behavior; on the other hand, he obtains useful information for its guidance in the implementation process. These parameters are chosen so that they can effectively be tuned at the wet-lab, i.e. they are effective biological tuning knobs. To show the proposed approach, the methodology is applied to the design of a well known biological circuit: a genetic incoherent feed-forward circuit showing adaptive behavior.

**Conclusion:**

The proposed multi-objective optimization design framework is able to provide effective guidelines to tune biological parameters so as to achieve a desired circuit behavior. Moreover, it is easy to analyze the impact of the context on the synthetic device to be designed. That is, one can analyze how the presence of a downstream load influences the performance of the designed circuit, and take it into account.

**Electronic supplementary material:**

The online version of this article (doi:10.1186/s12918-016-0269-0) contains supplementary material, which is available to authorized users.

## Background

Synthetic Biology is defined as the engineering of biology: the deliberate (re)design and construction of novel biological and biologically based parts, devices and systems to perform new functions for useful purposes [1]. As an engineering discipline, it emphasizes engineering principles and methodology in designing, constructing and characterizing biological systems to be applied in industrial, environmental and other applications. Currently, there still is a disparity between the ability to design systems and the one to synthesize them. This disparity can partly be attributed to a lack of well-characterized parts and methods for reliably and robustly composing parts into devices [2].

From the very beginning of Synthetic Biology, efforts have been made in order to characterize standard biological parts –i.e. DNA sequences encoding a function that can be assembled with other standard parts to form devices [3]. Yet, the roadmap to engineering biological systems is determined not by the biological parts but rather by how they interact [4]. Thus, both precise characterization and predictable part composition are essential for the efficient creation of sophisticated genetic circuits [5, 6]. In this context, developing frameworks for functional composition is a current challenge, the solution of which will allow biological components to be systematically, reliably, and predictably assembled into a functional device or system [2].

The systematic design of complex bio-circuits from libraries of standard parts relies on mathematical models describing the circuit dynamics. In this regard, modular modeling tools facilitate the mathematical representation of biological parts and their combinations, providing the description of the reactions which take place inside the different parts and the interfaces that connect them [7, 8]. Computer-aided (model based) methods and tools can be used to guide the design of synthetic biochemical pathways [9–11].

Several problems arise when building up biological devices by combining parts. First, composing different biological parts and devices together can be difficult, even if assuming a synthetic circuit structure has been properly designed to have a pre-specified dynamic behavior, because the desired input and output levels of a module are often unknown, difficult to measure quantitatively, or difficult to compare. Additionally, the ratio part/device performance may be altered due to the interaction of loads in the combined system, the so-called retroactivity [12]. Along with this, there is an ever-growing appreciation for biological complexity, which requires new circuit modeling and design principles to overcome barriers such as metabolic load, cross-talk, resource sharing, and gene expression noise [5, 13–15]. Finally, one must never forget the gap between computational (*dry-lab*) design, and *wet-lab* implementation. In practice, biological parts are subject to uncertainty. Circuit structure design and parameters tuning methods must cope with this uncertainty in the biological parts and context to narrow the gap.

To this end, the modular and systematic design of biocircuits, i.e. the systematic way of finding combinations of components from a library of standard parts allowing to optimally perform a pre-defined function, can be formulated using an optimization framework [16–18]. Indeed, it has been argued that Synthetic Biology is less like highly modular (or ‘switch-like’) electrical engineering and computer science, and more like civil and mechanical engineering in its use of models optimization of whole system-level stresses and traffic flow [5].

Advanced optimization-based methods, capable of handling high levels of complexity and multiple design criteria have been proposed for the modular and systematic structural design of biocircuits [19]. These new approaches combine the efficiency of global Mixed Integer Nonlinear Programming solvers with multi-objective optimization techniques [20, 21].

On the other hand, a natural approach to model-based tuning of synthetic circuits consists of the analysis of the effect of key parameters that can be used as tuning knobs in the experimental implementation. In this approach, selection of biological parts is understood as choice of the range of values of key parameters of the device that yield the desired dynamical behavior. A current challenge is to devise methods to provide the set of circuit parameters that satisfies a specified circuit behavior in a way that can be readily used for their wet-lab implementation [22]. Thus, for instance, in [23], the authors synthesize regulatory promoter libraries, characterize key parameters, and use them to guideline the construction of synthetic networks with different predicted input-output characteristics. Global sensitivity analysis is used in [16]. The sensitivity information is used to guide the selection of circuit components and thereby reduce the wet-lab implementation effort. In [24] the authors express the desired behavior as a functional cost index of the desired circuit trajectories. Then, the inverse sensitivity of the mapping between parameters and cost index is obtained after linearising the functional cost index around an initial value of the model parameters. This local inverse mapping is used to map a region of specifications into a one of parameters.

Although the specification of the desired dynamic of the circuit is most often naturally expressed as a multi-objective global optimization problem, this approach has not been used so far. Instead, current approaches define independent thresholds set a priori for each of the functional goals characterizing the desired behavior of the circuit. Then, global Monte Carlo-like approaches are used, sampling the parameters space and simulating the circuit time response. The result of these simulations is used to assess the circuit behavior, so as to profile the subset of the parameters space that result in circuit behavior fulfilling all thresholds. After this, some statistical post-treatment of the results is used, like clustering or correlation analysis or global sensitivity analysis, to draw conclusions between the distribution of the parameters, and the circuit behavior [25]. This Monte Carlo based approach has a huge computational cost. Given a defined search space in the parameters space, the Monte Carlo sampling does not ensure that a solution will be found, thus requiring a large number of samples to find solutions. This problem increases as the thresholds defining the acceptable circuit behavior are more stringent. On the other hand, the solution space obtained weighs, either equally or ad hoc, all the functional goals of the circuit. Thus, besides missing many possible optimal solutions, there may be little variability among the different solutions in the parameters space, making the statistical post-treatment less sensitive.

Feed-forward circuits have been used within this context as an important case-study. In [26] all three-node possible network topologies that present adaptive dynamical behavior are analyzed using function-topology maps based on Monte Carlo sampling in the parameters space. Using a simple enzymatic model, the authors draw design principles of adaptation circuits. They show that there are only two core solutions that achieve robust adaptation: negative feedback loops and incoherent feed-forward ones. In [27], the incoherent feed-forward adaptive enzyme network structure derived in [26], is used as case study. A method is proposed to make inferences on the contribution of individual parameters to specific components of the system. Classes of kinetic parameters are obtained that may correspond to varying strengths of enzymatic reactions that can be measured and classified experimentally. The authors show that, for a given network structure, certain types of values, or motifs, also exist for kinetic parameters in order to achieve specific system dynamics. Clustering in the parameters space to detect kinetic motifs, i.e. sets of parameters yielding desired circuit dynamics, is used in [25].

In this paper, to build a given functional device with desired dynamic behavior, we study the application of a multi-objective optimization design (MOOD) framework [28] to obtain a model-based set of guidelines for the selection of its biological parts. In MOOD all objectives are important, so all of them are optimized simultaneously. Thus, the solution rarely is unique, but a set of solutions called the *Pareto Front*. In this sense all solutions are Pareto-optimal and differ from each other in the trade-off of objectives that each one represents. Then, the design reduces to encode carefully the desired dynamics into the objectives and optimization problem itself in the MOOD [28]. As a result, the designer obtains qualitative regions/intervals of parameters along the Pareto Front giving rise to the predefined behavior of the circuit. Contrarily to the passive search for solutions of Monte Carlo-based approaches, the multi-objective optimization approach actively searches for all the optimal solutions as a first step. The MOOD framework also naturally provides a classification of the parameters along the Pareto front, by taking into account their effect on each of the goals. Moreover, this framework makes easy to analyze the impact of context on the synthetic devices to be designed. This can be done by just incorporating information about the relationship between the device and the context. In general, this means we only need to know where do we connect the device which is being designed and how we are connecting it. Including this information in the optimization problem, we obtain a qualitative region of parameters taking into account the effect of the context on the device.

The remaining of the paper is organized as follows. In Methods, the general framework, and the type-1 incoherent feed-forward (I1-FFL) circuit that will be used as case study, are presented. Next, in Results, the proposed methodology is applied to the I1-FFL case study, and the main findings for the circuit are described. Two typical application scenarios of the methodology are also considered. Finally, some discussion and general conclusions, both on the methodology and its results on the I1-FFL case study are drawn in Discussion and Conclusion sections.

## Methods

### Multi-objective optimization design framework

#### General workflow

Achieving a synthetic biological circuit fulfilling some behavioral specifications requires in practice an iterative process through three main steps: choosing a circuit structure capable to perform the desired behavior after the proper tuning of its parameters, tuning the circuit parameters, and validating the circuit with the selected tuned components. The use of models to solve the first two subproblems *in silico*, before attempting the wet-lab implementation to validate the circuit, reduces the wet-lab effort and speeds-up the design process. This work focuses on the second subproblem: *in silico* tuning of the circuit model parameters, so as to achieve the desired behavioral specifications.

First, a topology for the functional module or circuit is needed, capable to perform the desired behavior after the proper tuning of its parameters. This will provide the circuit model structure. Although currently there are no *catalogues* as such for functional modules, there is a vast literature in the systems biology area on network motifs producing a variety of dynamic behaviors [29]. Much work has also been done and is on-going on the design of circuits with various capabilities: repressilators [30], biomolecular concentration trackers [31], feedback regulation circuits [32], switchable genetic oscillators [33], etc. Many of the functional circuits that are being implemented in synthetic biology take advantage from well-established work in areas such as electronics and feedback control for the design of bistables, feedback and feedforward structures, switches, etc; see, for example, [26, 29, 34–39] and the references therein. Alternatively, one may find the potential circuit structure casting the problem as an optimization one, starting from coarse-grained models of the potential circuit structural components, and looking for the optimal circuit topology [19].

Models may have different degrees of detail. Our goal is to tune the model parameters using a degree of detail in the model amenable to serve as basis to provide guidelines for the experimental implementation of the circuit. That is, the parameters to be tuned should correspond to biological tuning knobs that can be modified experimentally [40]. Mass action kinetic models obtained from the set of biochemical reactions will be used for this purpose. These models can be reduced using singular perturbation methods (the so-called *quasi-steady state approximation*, QSSA) by neglecting the dynamics associated to fast binding reactions - e.g. RNA polymerase binding to DNA- and by taking into account the algebraic relationships among species resulting from conserved moieties [41]. The reduction process can be performed so that both the species in the reduced model are a subset of the original one [42, 43], and that the resulting aggregated parameters have a clear matching with experimental biological tuning knobs [44].

From this starting point, we can proceed to tune the model parameters so that eventually the circuit fulfills the behavioral specifications. We will consider the general case when a set of specifications is desired, thus leading to a multi-objective problem. A usual approach to face a multi-objective problem consists of building an aggregate function in order to assemble the design objectives in a unique index, normally by means of a weighting vector. This approach is followed for example in [25]. However, the solution obtained depends too much on the correct selection of the weighting factors, and it might not possibly reflect with enough clarity the designer’s preferences in relation with the desired balance of requirements. An alternative option is to use multi-objective optimization [45]. This is a natural choice to face this kind of problems. In multi-objective optimization all design objectives are important to the designer, so all of them are optimized simultaneously. Thus, the solution rarely is unique, but a set of solutions called the *Pareto Front*. In this sense all solutions are Pareto-optimal and differ from each other in the trade-off of objectives each one represents.

In order to successfully implement the multi-objective optimization approach, at least three fundamental steps are required [46], as depiced in figure depicted in Fig. 1:

1. the multi-objective problem (MOP) definition: defining the circuit behavioral specifications in a proper way.

**Fig. 1:**
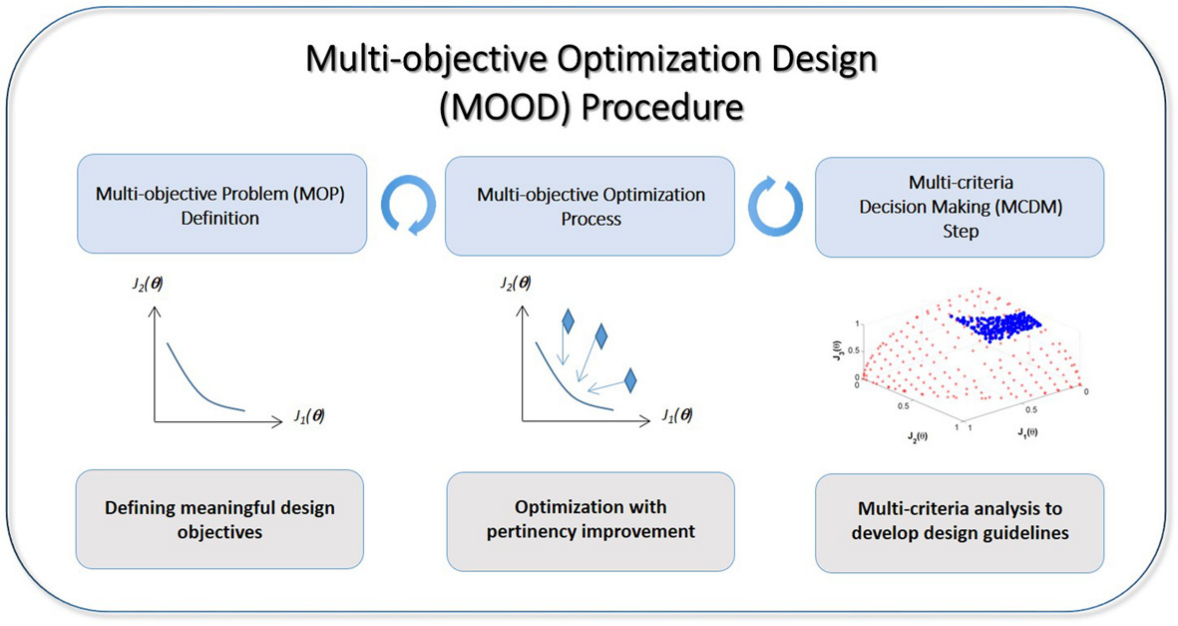
Steps for the multi-objective optimization design procedure
2. the optimization process: tuning the parameters using multi-objective global optimization (MOO).
3. and the multi-criteria decision making (MCDM) stage: obtaining tuning guidelines useful for the wet-lab implementation.

This overall multi-objective optimization design (MOOD) procedure enables to analyze design objectives trade-offs to implement a preferable solution [28]. Furthermore, it may provide a better understanding of the problem at hand by the so called process of *innovization* through optimization as stated by [47]. Next we describe each of the steps in detail.

#### Defining the circuit behavioral specifications

The starting point of the proposed methodology is the multi-objective problem definition, that is the specification of the desired dynamical behavior for the circuit to be designed. This can be done in several ways. From the designer’s point of view, specifying the circuit behavior in terms of the desired output signal profile for a given input signal profile is a natural approach [48]. The input signal is chosen as the one that is going to be used in working conditions, or as simple standard probing input-signals (e.g. step-like, sinusoidal, or pulse ones). Once the desired input-output relationship is defined, the set of circuit parameters achieving it can be obtained by optimization-based system identification [20]. This approach is useful for linear dynamical systems, as their time-response to these probing signals fully characterizes the circuit dynamical behavior. This is not the case for nonlinear circuits as the ones typically encountered in synthetic biology. Thus, the particular signal to be used in working conditions should be chosen. Yet, this may be very restrictive. Indeed, usually the input signal to a circuit will have varying characteristics. In the best case, it will belong to a given class (e.g. step-like signal with varying amplitude). Therefore, the dynamical behavior, i.e. the desired circuit time-response to a given input signal, is better given as a set of input-output performance indexes to be optimized.

Specifying the desired circuit behavior in terms of performance indexes to be optimized has many advantages. In the general case, the indexes will take the form of functionals mapping the circuit trajectories to the reals. Thus, consider a circuit with dynamics given by the model:

(1) $$  \begin{aligned} \dot{x} & = f(x,\theta)\\ 0 & = g(x,\theta) \end{aligned}  $$

where *x*∈*ℝ*^*n*^ is the state, *θ*∈*ℝ*^*p*^ the parameters, and function *g*(.) represents algebraic constraints in the system. The indexes can be expressed as:

(2) $$ J_{i}(\theta) = \int_{t_{0}}^{t_{f}} h(x(\tau,\theta),\tau) d\tau  $$

for some possibly time-dependent function *h*(.) of the system trajectories during a time interval of interest [*t*_0_,*t*_*f*_], being *i*=1…*n*_*i*_ is the number of indexes. These can be made valid for a whole class of input signals, may consider other signals in the circuit besides the input and output ones, robustness with respect to uncertainty in the circuit parameters can be included, etc. They will typically consider the desired performance at steady state (*precision*), and some measure of the quality of the transient. Proper definition of the optimization indexes representing the desired behavior is a key point. An incorrectly specified objective, not properly representing the actual desired behavior, will lead the optimization in a wrong direction, returning a parameter set that will give misleading design guidelines. Moreover, for the proper interpretation of results by the designer, one must pose meaningful design objectives.

#### Multi-objective parameters tuning

As mentioned above, representing the desired behavior will eventually lead to several objectives to be optimized. That is, the optimization problem will be a multi-objective one in the general case. Typically, some of the objectives will be in conflict, so a trade off among solutions is required. Ad hoc weighting of the different objectives may be used to transform the problem into a single-objective one [49]. Alternatively, thresholds on each of the objectives may be set in order to run multiple times a single-objective optimization. Instead, we address the problem as a truly multi-objective optimization design (MOOD) one.

The multi-objective optimization (MOO) process seeks to approximate the best parameters ${\theta _{P}^{*}}$ that give the best Pareto-front approximation ${J_{P}^{*}}$ [45]. Such search could be done through a random Monte-Carlo sampling in the decision variables space *θ* –the set of parameters determining our biological model–, followed by a filtering of the solutions in order to obtain the ${\theta _{P}^{*}}$ that defines the Pareto front approximation ${J_{P}^{*}}$. This could be a good option for problems with few decision variables. For problems with a large number of decision variables, as our case, it is more efficient to use an appropriate multi-objective optimization algorithm to approximate this solution.

We obtain the Pareto-optimal front of solutions via spMODE, a multi-objective optimization algorithm based on differential evolution [50, 51] implemented in Matlab, and available at Matlab Central^1^. The algorithm spMODE actively searches for all the solutions in the parameter space along the Pareto front. It:

- improves convergence by using an external file to store solutions and include them in the evolutionary process;
- improves spreading by using the spherical pruning mechanism [50];
- improves pertinency of solutions, i.e. getting interesting solutions from the designer’s point of view, by means of a basic bound mechanism in the objective space, as described in [52].

#### Obtaining tuning guidelines for implementation

After the multi-objective optimization, a set of solutions is obtained: values for the kinetic parameters that represent a trade-off between the objectives. Then, the final step is to obtain tuning guidelines to select the values of the kinetic parameters of the model and correspondingly cues for the implementation of the circuit in the wet-lab. In this work we present two alternatives for this last step: a semi-automated one based on an optimized clustering of the solutions, that is, providing some guinelines; and a second one, in case the implementation needs more insight allowing to learn more about the problem, based on the visualization of the Pareto front and set using suitable tools, thus, providing a guidance with this information.

In the first alternative, qualitative instructions for the wet-lab implementation are extracted from these solutions. The kind of information extracted is in the form of qualitative levels for the kinetic parameters that can be commonly modified in the wet-lab, for instance:

- Plasmid copy number. It can be tuned by selecting the appropriate replication origin of the plasmid.
- Promoter strength. It can be modified by selecting the appropriate promoter with predicted strength; for example from the Anderson Promoter library [53] available at the iGEM Parts Registry.
- Ribosome Binding Site strength. It is one of the easiest parameters to tune in the wet-lab using, for instance, RBS libraries, the RBS Calculator from Sallis Lab [54], or nucleotides repetition [55].
- Protein degradation rate. It can be tuned globally by changing the growth rate of the microorganism. It can also be tuned by adding a protein degradation tag to include the protein in an active degradation pathway.

In order to facilitate the obtention of the guidelines, a hierarchical clustering is performed with the solutions (also using a Matlab script, see Additional files 1 and 2), including the values of the objectives and also the kinetic parameters of each solution. This process is achieved by using a cluster tree based on the Euclidean distance among the vectors containing the attained values of the objectives for all points along the Pareto front. The distance among clusters is obtained by means of the weighted center of mass distance. Then we set the number of clusters in an iterative manner from ten to two, and in each iteration we perform a Kruskal-Wallis [56] test (which is the non-parametric equivalent of the one-way analysis of variance ANOVA) to study the correlation between the kinetic parameters and the clusters. With this process the optimal number of clusters is selected by choosing the one that maximizes the number of significantly correlated parameters with the clusters. Each one of the resulting correlated parameters has different value ranges in each one of the clusters which represents a guideline for this parameter. For example it can range around low values (with respect to the initial interval for that parameter) for some clusters and high values for other clusters. This parameters are particular guidelines for each cluster.

For the parameters that do not exhibit a significative correlation, its optimized range is also checked against the initial interval given to the optimizer. If the ranges are different this means the optimization process found an optimal range for the parameter, but general to all the clusters. This parameters are general guidelines for optimality.

For the second alternative, it is accepted that visualization techniques are valuable in order to analyze the trade-off among competing objectives. Such visualization and analysis is not a trivial task when the number of objectives is larger than three and/or the number of decision variables in the Pareto set is large, like in our case. Several tools are available, but in any case, some desirable characteristics are useful to perform such analysis. The first of them are concerned with the practical aspects of the analysis:

- It must enable design alternatives comparison (analyze different solutions).
- It must enable design concepts comparison (analyze different Pareto front approximations).

Others are related to subjective aspects of the visualization:

- Completeness: all relevant information should be contained in the visualization.
- Persistence: all the relevant information should be retained in the designer’s mind.
- Simplicity: the visualization should be easily understandable.

In this work we use the visualization tool Level Diagrams (LD) [57, 58], which has a freely available implementation for designers: LD-Tool^2^. LD-Tool allows to correlate design objectives with decision variables. It classifies the calculated optimal parameters ${\theta }_{P}^{*}$ with respect to each objective *J*_*q*_(*θ*) normalized with respect to its minimum and maximum value. A graph for each objective is displayed (see Additional file 1: Figure S1), where the Y-axis is the p-norm $\|\hat {{J}}({\theta)}\|_{p}$ of the objectives vector, and the X-axis corresponds to the objective value or decision variable depending on the case. A second graph displays $\|\hat {{J}}({\theta)}\|_{p}$ with respect to each decision variable. These characteristics make it helpful in order to propagate the information from clustering between design objectives space and decision variables space. Thus, a given solution will have the same value -*y* in all graphs. As it is, LD enables the alternative and design concept comparison. In order to also incorporate the information obtained in the clustering, the y-axis of the LD plot is modified to show the membership of a solution to a cluster, therefore, improving completeness for our problem. And this is coded also in the color of the points in all the graphs, improving persistence and simplicity. This correspondence of colors helps to evaluate general tendencies along the Pareto front and compare solutions according to the clusters they belong to. Additionally, with the aim of improving simplicity and completeness, the dynamic response of species from the model is ploted using the same color code. To sumarize, this step consists in first the clustering of the solutions and then: *For the guidelines*Study correlations between the parameters and the clusters and obtain guidelines. *For the guidance to help manual decision making*Visualization of the Pareto Front and Pareto Set of the clustered solutions to obtain more insight and learn about the specific problem.

All this step is performed in matlab scripts (see Additional files 1 and 2 for a description and the scripts respectively)^3^.

Finally, it is interesting to note that the selection of the preferable solution according to designer’s criteria, or equivalently the extraction of qualitative levels for the parameters, takes place in an *a-posteriori* multi-criteria analysis of the Pareto Front approximation, and it is in general computationally cheap in comparison with the multiobjective optimization step.

### Incoherent type 1 feed-forward loop (I1-FFL)

**Fig. 2:**
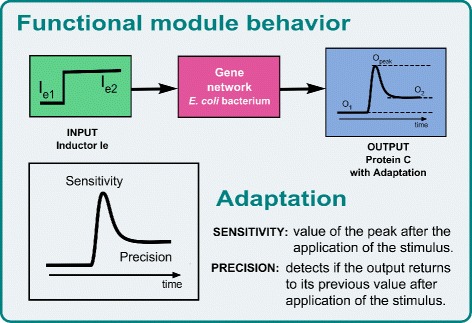
Input-output adaptive behavior. Adaptation is an important property of biological systems, related to homeostasis. After an input stimulus the output signal responds by first quickly reaching a peak value, after which it returns to its previous value even if the stimulus persists

**Fig. 3:**
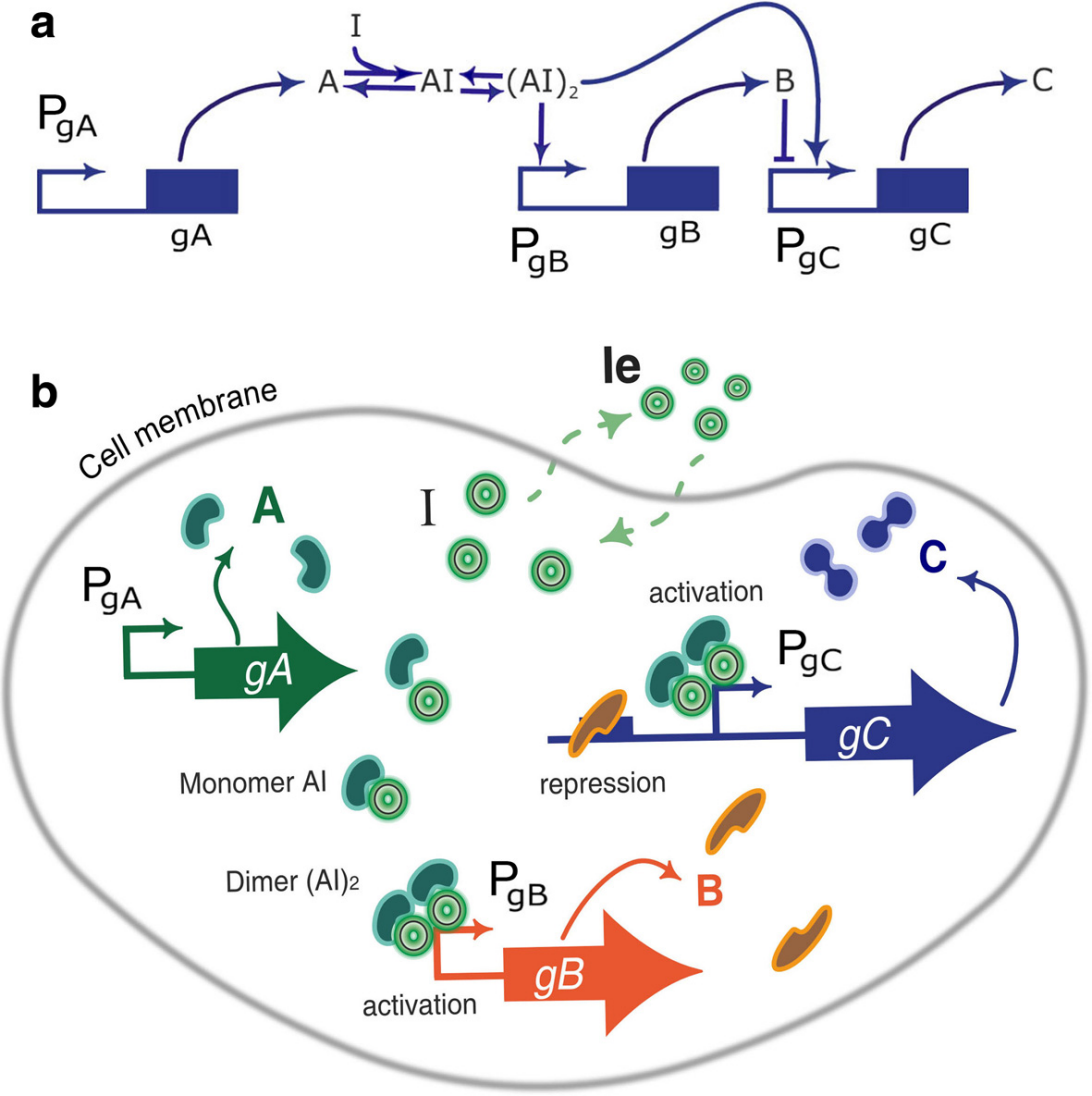
Three-node incoherent type 1 feedforward loop. **a** Gene gA produces the protein A, which forms a dimer with the inducer I. The dimer activates both genes gC and gB. In turn, the product of gB represses gC. **b** Representation of a cell incorporating an incoherent feedforward loop synthetic circuit

Adaptation is an important property of biological systems, linked to homeostasis [29], and to the generation of responses that depend on the fold-change in the input signal, and not on its absolute level [59]. It is defined as the particular ability of biological circuits to respond to a change in its input and return to the value it had prior to the stimulus, as depicted in Fig. 2. Due to its relevance, in the paper we will use a genetic circuit showing adaptation to illustrate the proposed approach. Circuit topologies giving rise to adaptive behavior have been extensively studied [29]. Different three-node topologies are possible [26]. Among them, the incoherent type 1 feed-forward loop structure (I1-FFL) is one the most common network motifs. Different implementations are possible, including enzyme reaction networks [26, 27], gene networks [34, 60] and in vitro transcriptional networks [61]. In the gene network case, a protein A acts as a transcription factor and activates expression of two downstream genes B, and C. In turn protein B represses expression of gene C. Figure 3a depicts the genetic synthetic circuit. To introduce a step-like input signal to the circuit, we consider the addition of an external chemical inducer *I*, that diffuse from the extracellular culture inside the cell. Most of these inducers undergo an heterodimerization, i.e. the inducer binds to one of the circuit species thus effectively providing an input to the circuit. Most of them subsequently dimerize. We have used a model that captures both phenomena. The protein A, product of gene A, bounds to the inducer I, forming a monomer *A*·*I* which in turn dimerizes. The dimer (*A*·*I*)_2_ is the transcription factor that activates expression of gene C directly, and represses it indirectly via activation of the repressor B. As a result, when a signal causes node A to assume its active conformation, C is produced, but after some time B accumulates, eventually attaining the repression threshold for the gene C promoter.

We model the designed genetic circuit using a deterministic approach and taking into account the key regulatory interactions between the main biochemical species present in the genetic circuit: proteins *A*,*B*, and *C*, and inducer *I*. In our gene synthetic circuit (see Fig. 3b), the circuit comprises a gene gC under the control of the promoter *P*_gC_. The concentration of protein *C* is considered to be the circuit output signal. Expression of *C* is activated by the dimer (*A*·*I*)_2_ that acts as transcription factor for the hybrid promoter *P*_gC_, and it is repressed by protein *B*. The dimer (*A*·*I*)_2_ also acts as transcription factor activating the promoter *P*_gB_. Protein *A* is constitutively expressed, and bounds to the inducer *I*. The inducer can passively diffuse across the cell membrane. Though the input signal to the circuit is the intracellular inducer concentration *I*, the experimental input signal is the external application of the inducer in the broth *I*_*e*_.

Starting from a complete model based on mass action kinetics (See Additional file 1, 1.I1-FFL Model) we obtained the reduced deterministic model (3).

(3) $$\begin{array}{@{}rcl@{}} {\begin{aligned} &\dot{x}_{1} = k_{mA} C_{gA} - d_{mA} x_{1}\\ &\dot{x}_{2} = k_{pA} x_{1} - d_{A} x_{2} - k_{2} x_{2} x_{3} + k_{-2} M \\ &\dot{x}_{3} = -k_{2} x_{2} x_{3} + k_{-2} M + k_{d} (x_{9} - x_{3}) - d_{I} x_{3} \\ &\dot{x}_{4} = k_{3} M^{2} - k_{-3} x_{4} - d_{AI2} x_{4} \\ &\dot{x}_{5} = K_{mB} \: C_{gB} \frac{x_{4}}{\upgamma_{1} + x_{4}} - d_{mB} x_{5} \\ &\dot{x}_{6} = k_{pB} x_{5} - d_{B} x_{6} \\ &\dot{x}_{7} = K_{mC} C_{gC} \frac{x_{4} + \beta_{1} \gamma_{4} x_{6} + \beta_{2} \gamma_{5} x_{4} x_{6} + }{\upgamma_{2} + \upgamma_{3} x_{4} + \upgamma_{4} x_{6} + \upgamma_{5} x_{4} x_{6}} - d_{mC} x_{7} \\ &\dot{x}_{8} = k_{pC} x_{7} - d_{C} x_{8}\\ &\dot{x}_{9} = K_{cells} k_{d} \left(- x_{9} + x_{3} \right) - d_{Ie} x_{9}\\ & M = - \frac{d_{AI} + k_{-2}}{4 k_{3}} + \frac{1}{4 k_{3}} \sqrt{(d_{AI} + k_{-2})^{2} + 8 k_{3} (k_{2} x_{2} x_{3} + 2 k_{-3} x_{4})} \end{aligned}} \end{array} $$

where *M* is the monomer concentration, and $K_{cells} = \frac {V_{cell}N_{cells}}{V_{medium}}$ the volumes relationship required to take into account the concentration outside the cells. Note the transport term (*x*_3_−*x*_9_), depends only on the difference of the concentrations inside and outside the cells. The *K*_*cells*_ constant reflects the amount that goes out (or in, depending on the sign) from all the cells into the extracellular volume. In the simulations we used *V*_*cell*_=1×10^−15^*L*, which is the typical volume of an *E. coli* cell, we considered *N*_*cells*_=2.4×10^8^ cells/mL∗0.18 mL which is the number of cells in a 180 *μL* culture with *OD*=0.3 placed in a well containing *V*_*medium*_=180*μL* of culture medium. Table 1 shows the species and their corresponding symbols.

**Table 1:** List of variables used in the reduced model

Variable	Description	Units	Symbol
*x*_1_	mRNA _gA_	nM	mA
*x*_2_	A protein	nM	A
*x*_3_	Inducer	nM	I
*M*	A ·I monomer	nM	A ·I
*x*_4_	(A ·I)_2_ dimer	nM	(A ·I)_2_
*x*_5_	mRNA_gB_	nM	mB
*x*_6_	B protein	nM	B
*x*_7_	mRNA_gC_	nM	mC
*x*_8_	C protein	nM	C
*x*_9_	Extracellular inducer	nM	I_*e*_

Model (3) has nine differential equations plus one algebraic equation (M) and 26 parameters, described in Table 2. Although from the model reduction process more algebraic relations were obtained, (See Additional file 1, 1.I1-FFL Model), they are simple enough to be directly replaced into the model.

**Table 2:** Parameters of the reduced model

Parameter	Description	Value	Unit
$C_{{g_A}}, C_{{g_B}}, C_{{g_C}}$	gA, gB, gC copy number	-	adim.
$k_{{m_A}}, k_{{m_B}}, k_{{m_C}}$	gA, gB, gC transcription rate	-	min ^−1^
$d_{{m_A}}, d_{{m_B}}, d_{{m_C}}$	m _*A*_, m _*B*_, m _*C*_ degradation rate	0.3624	min ^−1^
$k_{{p_A}}$	m _*A*_ translation rate	80	min ^−1^
$k_{{p_B}}, k_{{p_C}}$	m _*B*_, m _*C*_ translation rate	-	min ^−1^
*d*_*A*_	A degradation rate	0.035	min ^−1^
*d*_*B*_,*d*_*C*_	B, C degradation rate	-	min ^−1^
*k*_*d*_	inducer diffusion rate	0.06	min ^−1^
*k*_2_, *k*_3_	(AI) and (AI) _2_ association rate	0.1	min ^−1^
*k*_−2_	(AI) dissociation rate	20	min ^−1^
*k*_−3_	(AI) _2_ dissociation rate	1	min ^−1^
*γ*_1_	gB promoter Hill constant	-	nM
*γ*_2_	gC promoter coefficients	0.2	nM
*γ*_3_,*γ*_4_,*γ*_5_	gC promoter coefficients	-	adim, adim, nM ^−1^
*β*_1_,*β*_2_	gC promoter basal expression coefficients	0.05	adim, nM ^−1^
*d*_*I*_,*d*_*Ie*_	inducer degradation rate	0.0164	min ^−1^
*d*_*AI*_,*d*_*AI*2_	(AI), (AI) _2_ degradation rate	0.035	min ^−1^

An SBML implementation of this model was deposited in BioModels [62] and assigned the identifier MODEL1511290000. This implementation is not part of the multi-objective optimization design procedure, although it was included for completenes and is intended to be used separately. The implementation as matlab scripts is in the Additional file 2, and will be available in Matlab Central.

For the simulations implemented in the next section, the values in Table 3 are selected for the kinetic parameters that are not considered decision variables.

**Table 3:** Parameters of the reduced model selected for optimization

Parameter	Wet-lab implication
$ k_{m_{B}}C_{g_{B}}, k_{m_{C}}C_{g_{C}}$	Promoter strength and Plasmid origin of replication
$k_{p_{B}}, k_{p_{C}}$	RBS Strength
*γ*_1_,*γ*_3_,*γ*_4_,*γ*_5_	Mutations in promoter sequence
*d*_*B*_,*d*_*C*_	Degradation tag sequence

## Results

Using the presented framework we considered its application for tuning the kinetic parameters of the I1-FFL circuit to achieve adaptation behaviour. The idea is to apply the three steps of the MOOD considering the I1-FFL model presented in the previous section. This way, the implementation of the MOOD procedure will be clarified by an example. Later we will show two scenarios related with the wet-lab implementation and usability of the guidelines obtained.

### I1-FFL tuning using MOOD framework

#### Multi-objective problem (MOP) definition

The first step of the MOOD framework is to formulate the circuit specifications as design objectives to be optimized. Recall the desired input-output behavior for the I1-FFL circuit, depicted in Fig. 2. Let *θ* denote the following subset of parameters selected for optimization from the reduced model (3):

Two basic objectives can be considered for this circuit [25, 26, 60, 63]:

- **Sensitivity:** after input stimulation, a clear transient peak value is desired for the output. Sensitivity can be defined in relative terms as the relationship between the input and output variation during the transient. In our case, we define sensitivity as the ratio between the absolute total variation of the output signal –the C protein concentration *x*_8_–, and the variation of the input signal –the external inducer *x*_9_.
- **Precision:** after the peak transient, the output must go back to its value previous to circuit stimulation. Thus, precision can be defined as the inverse of the normalized output error. The lower the steady state error, the higher the precision.

Our design objectives can be mathematically expressed by means of the indexes:

(4) $$\begin{array}{@{}rcl@{}} \begin{aligned} J_{1}({\theta}) &= \frac{2 \left(x_{9}(t_{f}) - x_{9}(t_{0}) \right) }{\int_{t_{0}}^{t_{f}} |\frac{d x_{8}}{dt} | dt} \\ J_{2}({\theta})&= \frac{x_{8}(t_{f}) - x_{8}(t_{0})}{x_{9}(t_{f}) - x_{9}(t_{0})} \end{aligned} \end{array} $$

where *t*_*f*_ is the time length of the experiment. The input stimulus is applied at *t*_0_.

Sensitivity is the inverse of *J*_1_(*θ*). Notice the total absolute variation of the C protein concentration is obtained as half the accumulated absolute value of the time derivative of *x*_8_. The lower *J*_1_(*θ*) (larger output peak w.r.t. input variation), the higher the sensitivity.

Precision is the inverse of *J*_2_(*θ*), i.e. the inverse of the ratio between the variation of the C protein concentration between *t*_0_ and *t*_*f*_, and the variation of the external inducer concentration between *t*_0_ and *t*_*f*_. If the C protein concentration *x*_8_ at time *t*_*f*_ is the same as the initial one at time *t*_0_, precision is infinite.

Note that both objectives are defined as the inverses of Sensitivity and Precision in order to use them in the minimization problem as it is the standard for optimization problems [46].

Additionally, other objectives could be considered. For instance, fulfillment of constraints on the species. In our case, in order to obtain realistic solutions regarding the values of protein B concentration, its absolute total variation was taken into account as a constraint. This can be expressed as:

$$\begin{array}{@{}rcl@{}}  P({\theta}) &=& \int_{t_{0}}^{t_{f}} \left|\frac{d x_{6}}{dt} \right| dt, \end{array} $$

We considered the constraint:

(5) $$  1 < P({\theta}) < 10000  $$

To make the precision higher (that is, low output error) the easiest option is to have very high values of protein B concentration, which acts as repressor of protein C. To avoid this unrealistic solution, it is possible to make the concentration of protein B to have an upper bound. In the case of not having this restriction, the solutions may have higher precision at the cost of unrealistically high values of protein B concentration. The restriction penalizes this fact and drives the search to a different region of the parameter space (going away from this undesired region, the one corresponding to high values of protein B).

Another relevant issue is the definition of limits for *J*_1_(*θ*) and *J*_2_(*θ*) beyond which we consider that precision and sensitivity degrade to such an extent that we cannot talk about adaptive behavior anymore [26]. This is the so-called *pertinency* range of the objectives. The limits established in this work are: *J*_1_(*θ*)∈[ 1×10^−3^, 200], and *J*_2_(*θ*)∈[ 1×10^−4^, 20].

Finally, we look for the set of values for the 10 decision variables *θ* that optimize both objectives. Yet, precision and sensitivity are conflicting objectives. So a trade-off must be reached. Therefore, our problem can be formulated as a multi-objective problem (MOP):

(6) $$\begin{array}{@{}rcl@{}}  \begin{aligned} \min_{{\theta} \in \mathfrak{R}^{10}} {J(\theta)} & = & \left[J_{1}({\theta}), J_{2}({\theta})\right] \in \mathfrak{R}^{2}&\\ \textrm{subject to:} & & \text{Eq.~(3)}&\\ & & 1\times10^{-3} < J_{1}({\theta}) < 200\\ & & 1\times10^{-4} < J_{2}({\theta}) < 20\\ & & 1 < P({\theta}) < 1\times10^{5} \end{aligned} \end{array} $$

#### Multi-objective optimization

As a second step we carried out the dynamic optimization of (6) using the multi-objective differential evolution spMODE genetic algorithm described in Subsection Multi-objective parameters tuning. Starting from an initial random population of candidate solutions, we set 15.000 iterations as the maximum number of evaluations of the objective functions. We obtained a Pareto front containing 33 solutions that achieve adaptation, together with the Pareto set containing the kinetic model parameters corresponding to the Pareto front solutions (see Additional file 1: Table S3). These solutions show, as expected, a trade-off. Solutions range from high sensitivity (low values of *J*_1_) and low precision (high values of *J*_2_) ones to low sensitivity (high values of *J*_1_) and high precision (low values of *J*_2_) ones. Note in all cases these solutions are the optimal ones, in the sense of Pareto.

**Fig. 4:**
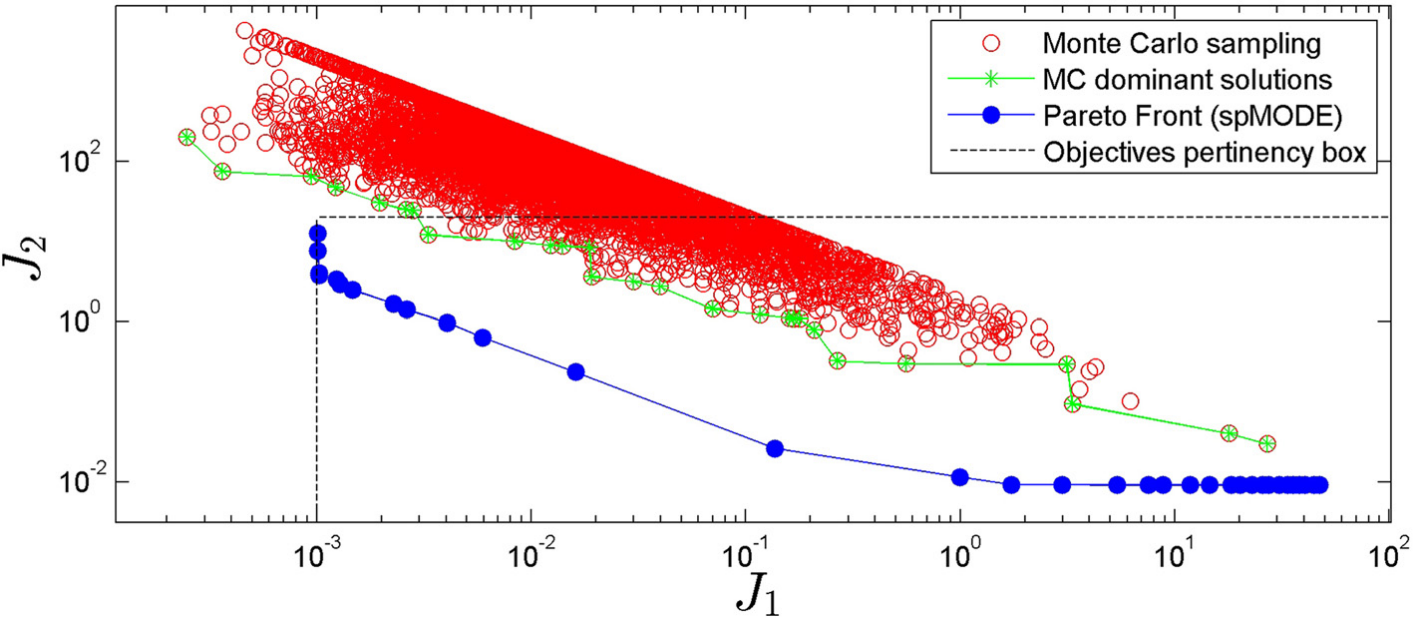
Pareto Front comparison. Pareto Front representation for *J*_1_ and *J*_2_ obtained with the spMODE algorithm for the MOO (blue line). Monte-Carlo random sampling results are colored in red and the dominant solutions are in green. The time response of the C protein concentration for three representative points are shown

A Monte-Carlo sampling (MCS) and a Latin Hypercube sampling (data not shown) with the same computational cost were performed for the sake of comparison. In both cases, the solutions must be selected with a dominance filter so as to detect the ones actually fulfilling the constraints and yielding adaptive dynamics [25]. Note this functional association step is not required in our approach, as the optimal sets of parameters obtained already correspond to functional ones. From the functional solutions obtained with these sampling techniques, we approximated the corresponding Pareto front. Figure 4 shows the results obtained. The Pareto front obtained from the MCS (dominant solutions in green) covers a larger region of the objectives space, but outside of our region of interest (pertinency box), and it is far away behind the optimal one obtained with spMODE.

#### Obtaining guidelines for the implementation

The third step is to obtain guidelines and guidance for the implementation of the circuit. To obtain the guidelines, the solutions gathered from the optimization were clustered hierarchically in an agglomerative tree (see Matlab code in Additional file 1) and the optimal number of clusters obtained with the procedure explained in the Methods section. The guidelines obtained are shown as intervals in the next Table.

As result we can put into words the following general guidelines, which are necessary for achieving adaptation:

- *d*_*B*_: Degradation rate of protein B, has to be the lowest possible in all the cases.
- *Kp*_*B*_: The RBS strength of gene B has to be the lowest possible in all the cases.
- *γ*_1_: The promoter strength (activation strength) has to be high in general, but it does not has an apparent effect.
- *γ*_3_: The hybrid promoter strength (activation strength), has to be the lowest possible in all the cases.

Depending on whether high sensitivity or high precision are chosen, specific guidelines (see Table 4) can be given for the tuning knobs to be modified in the wet-lab so as to tune the behavior of the circuit:

■■■ **High Sensitivity Strategy:**
  *Km*_*C*_*CgC* and *Kp*_*C*_: increasing values of the promoter strength and plasmid copy number of gene C, and the RBS Strength of gene C, lead to increasing values of sensitivity (higher peak values). These are tuning knobs for sensitivity.

**Table 4:** Design guidelines. Each one of the optimized parameters is either a general guideline for all clusters, or is a trade-off control tuning knob for a specific cluster

Parameter	Initial parameter range	Design guideline		
		General guideline	Cluster 1	Cluster 2
kmACgA ^∗^	[1 200]	–	[1 171.91]	1
kmBCgB	[1 200]	–	1	[1 200]
kmCCgC	[1 200]	–	[1 171.91]	1
k_pB_	[1 100]	1	–	–
k_pC_	[1 100]	–	[1 15.68]	1
d_B_	[0.01 0.3]	[0.01 0.0792]	–	–
d_C_	[0.01 0.3]	–	[0.2784 0.3]	0.3
*γ*_1_	[50 200]	[78.93 200]	–	–
*γ*_3_	[1e-4 0.5]	–	[1e-4 0.013]	[1e-4 0.0141]
*γ*_4_	[5e-4 5]	–	[5e-4 1.4424]	[0.0697 5]
*γ*_5_	[1 100]	–	[1 9.2546]	[12.125 100]

^*^kmACgA Is the same as kmCCgC as the are physically in the same plasmid
  *d*_*C*_: degradation of protein C has to be slightly lower for high sensitivity.
  *γ*_4_ and *γ*_5_: Hybrid promoter strengths (repression, and activation - repression cross combined strength), should be kept low.
  *Km*_*B*_*CgB*: Promoter strength and plasmid copy number of gene B, must have low values.
■■■ **High Precision Strategy:**
  *Km*_*B*_*CgB*: Promoter strength and plasmid copy number of gene B, is a tuning knob for Cluster 6, increasing precision proportionally to its value.
  *γ*_4_ and *γ*_5_: Increasing values of the hybrid promoter strengths lead to increasing values of precision (lower error).
  *Km*_*C*_*CgC* and *Kp*_*C*_: Promoter strength and plasmid copy number of gene C, and the RBS Strength of gene C, keep them low.
  *d*_*C*_: degradation of protein C has to be high.

The results show that the value of *d*_*C*_, i.e. the degradation rate of the C protein, is a key parameter to correctly achieve adaptation. With high values of this parameter, the concentration of the C protein will to return faster to its original level.

Some parameters *γ* in the hybrid promoter of protein C are also forced to take certain values for the system to attain the adaptive behavior. In particular, it is interesting to notice that the repression strength, parameter *γ*_4_ plays an important role, which is in agreement with the analysis in [34], where a mutation was performed on the hybrid promoter so as to affect the same parameter.

In the case the designed needs more insight, we provide the tools for visualization to allow a proper decision making procedure and selection of the appropriate parameters for the design.

**Fig. 5:**
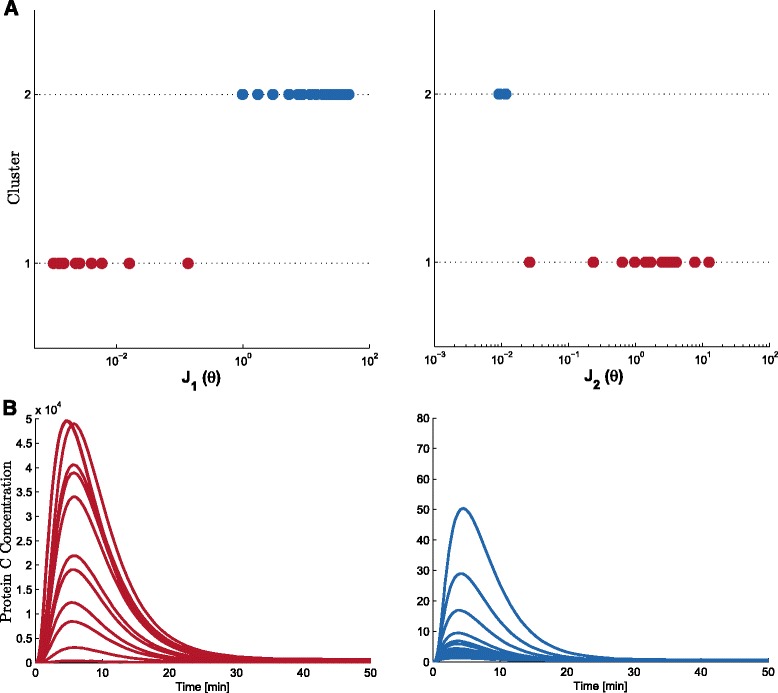
Pareto front representation in the cluster-modified LD tool. **a** Value of the objectives *J*_1_ and *J*_2_ for each solution where each cluster is identified by a different color. Clusters range from high sensitivity-low precision (*red*) to low sensitivity-high precision ones (*blue*). **b** Time courses of protein C concentration for the different solution in the clusters

**Fig. 6:**
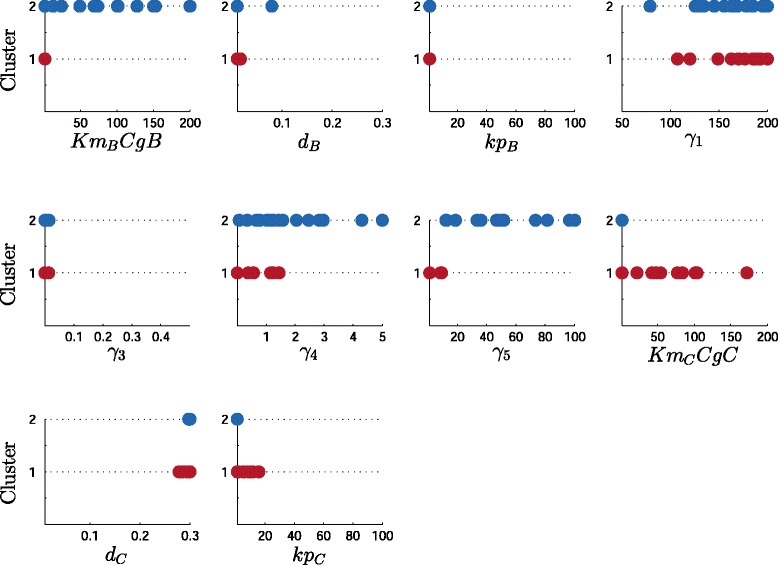
Representation of the Pareto set. Cluster-modified LD representation for decision variables (kinetic parameters) in the High Sensitivity Strategy (cluster 1, red dots) and in the High Precision Strategy (cluster 2, blue dots)

The Pareto front together with the time response of the C protein concentration for each point are shown in Fig. 5. Clusters range from high sensitivity and low precision (cluster 1) to low sensitivity-high precision ones (cluster 2). In Fig. 6 the Pareto set is depicted the value of each parameter and its membership to the corresponding cluster. This way is easy to directly find the implication of each parameter in the design. After the analysis of the Pareto set plot it is possible to find: on the one hand, parameters *d*_*B*_,*Kp*_*B*_ and *γ*_3_ have uniform (and tight) values for both clusters and *γ*_1_ has a uniform and wide range of values also for both clusters. On the other hand, we find basically two different strategies: one for high sensitivity (clusters 1, with red color, in Fig. 6) which changes parameters in gene C (*Km*_*g*_*CgC*,*kp*_*C*_ and less *d*_*C*_), and another one for high precision (clusters 2, blue colors, in Fig. 6) which changes parameters in gene B and in the hybrid promoter (*Km*_*B*_*CgB*,*γ*_4_ and *γ*_5_).

In the Additional file 1: Figures S3 and S4 the original Level Diagrams of the Pareto front and set are shown, in case the designed needs more information and insight for the guidance of its multi-criteria decision-making.

### Application scenario I: selecting parameters for an implementation

As a proof of concept, and also to validate the guidelines obtained for the I1-FFL we proceed as we would do in the wet-lab. Let us suppose we have two implementations obtained with the guidelines proposed in this work: one designed with the High Sensitivity Strategy (Case A) and another one with High Precision Strategy (Case B).

**Fig. 7:**
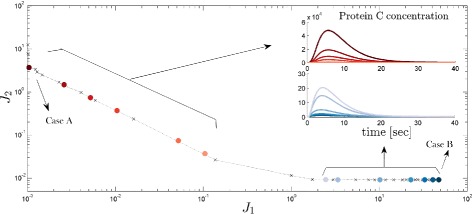
Application scenario I Pareto Front in blue line connected dots. A. Dots with reddish color are obtained when using the RBS strength of gene C as a trade-off tuning knob and represented by modifying *k* *p*_*C*_∈ [ 5 0.05] starting at the extreme solution. Notice, that decreasing only *k* *p*_*C*_ it is possible to increase the sensitivity, almost without losing optimality (without getting away from the Pareto front). Inset shows the time course of protein C. As expected, sensitivity of the solution is increased, i.e. the peak of protein concentration after stimulus is higher. B. Dots with blueish color are obtained when using the promoter strength and plasmid copy number gene B by modifying *K* *m*_*B*_ *C* *g* *B*∈ [ 200 1]

The Case A is a solution with low precision, but high sensitivity as it belongs to cluster number 1. It is located in the low extreme of *J*_1_, and in the high end of *J*_2_ in Fig. 5. For this design will use the High Sensitivity Strategy and we will choose, for example, *kp*_*C*_ as a tuning-knob. Changing the value of this parameter will affect the position of the solution in the Pareto front. Although, moving exactly along the Pareto front requires modifying more parameters as shown in the guidelines before, we can see (by looking at the reddish dots in see Fig. 7) how the initial chosen solution moves almost on top of the Pareto front. This shows that the obtained guidelines are robust so that we can use the selected parameter as a tuning knob in the wet-lab implementation.

Also, starting from the high precision implementation (Case B), we show how changing one of the tuning knobs from our High Precision Strategy (*Km*_*B*_*CgB* for example) one can almost move along the Pareto front and obtain higher sensitivity solutions without losing precision, as shown by the blueish dots. In the insets of Fig. 7 is possible to see the temporal behavior of the obtained solutions.

Conversely to this, changing values of key parameters like *d*_*C*_ completely destroy the adaptation behavior independently of the selected solution (see Figure S2 in Additional file 1).

### Application scenario II: output robustness analysis

This framework is also useful to analyze the output performance of the designed functional device when connecting it to other devices.

**Fig. 8:**
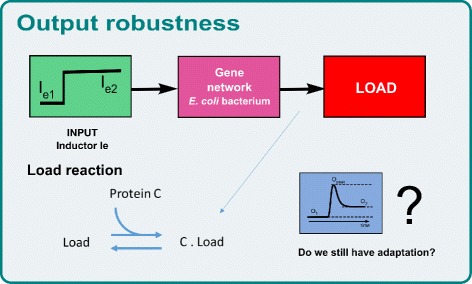
Application scenario II Depiction of the incorporation of information on the context. Connecting our module to a load

Here we will use a simple binding reaction as a load to demonstrate the procedure (see Fig. 8). This is one of the most common types of load. For example, the protein C could be a transcription factor and bind to a promoter region in the DNA. The next equations model this load binding reaction:

(7) $$\begin{array}{@{}rcl@{}} \begin{aligned} \dot{x}_{8} & =& k_{pC} x_{7} - d_{C} x_{8} -K_{1} x_{8} x_{10} + K_{2} x_{11} \\ \dot{x}_{10} &=& -K_{1} x_{8} x_{10} + K_{2} x_{11} \\ \dot{x}_{11 }&=& K_{1} x_{8} x_{10} - K_{2} x_{11} \\ \end{aligned} \end{array} $$

where *x*_10_ represents the empty load species (e.g. an unbound promoter or protein), and *x*_11_ represents the complex C bound to the load species. *K*_1_ and *K*_2_ are the binding constants. For this case we used *K*_1_=40 *nM*^−1^*min*^−1^, and *K*_2_=20 *min*^−1^, which correspond to a mildly fast binding. We chose the initial condition *x*_10_(*t*_0_)+*x*_11_(*t*_0_)=800 *nM*. Since we did not consider degradation terms in (7), this initial condition represents the total amount of available load species.

**Fig. 9:**
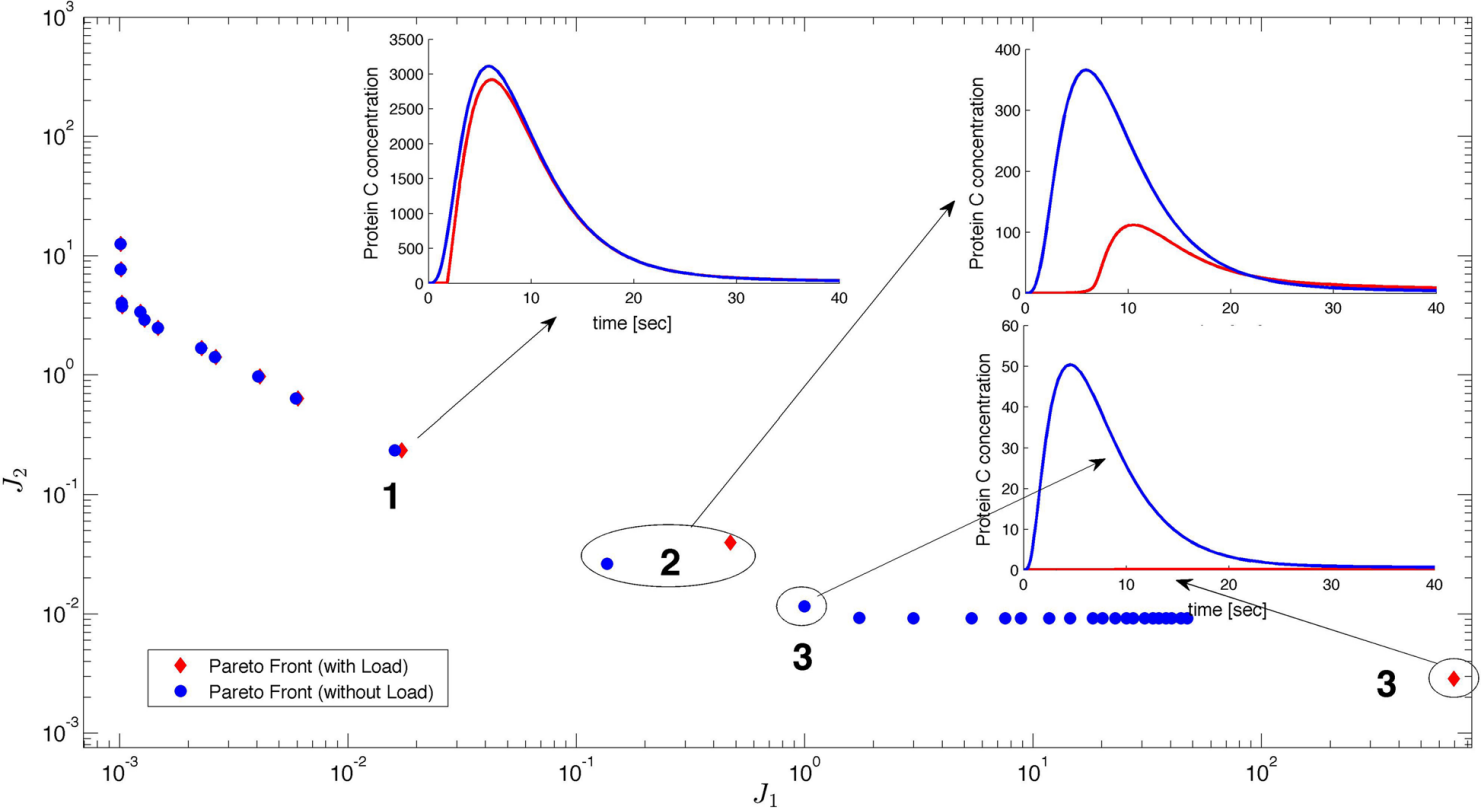
Application scenario II Pareto front of the functional module without load (*blue circles*) and with load (*red diamonds*). Inset: temporal responses of the solutions 1, 2 and 3 with (*red line*) and without load (*blue line*)

In Fig. 9, the Pareto front of the loaded device is shown in red colored diamonds, and the original Pareto front in blue circles. Notice that the analysis needs to be performed only along the Pareto front solutions. Thus, it is computationally very efficient. As we see for the I1-FFL circuit, solutions with low sensitivity are more affected by the load effect at high values of *J*_1_, i.e. lower peak values of C protein. This happens when the concentration of C is in the order of 800 *nM*, which is the total amount of load species concentration in this example.

Finally, in the inset of Fig. 9, the loaded time courses of the protein C concentration after stimulus (red line) are shown and compared with the original ones (blue line) for values of the parameters corresponding to solutions 1, 2 and 3. As we see, solution 1 is practically not affected; but solution 2 is affected considerably. Finally, solution 3 is way out from is location and actually looses adaptation behavior. Consequently, it is possible to use this framework to evaluate the output performance of our designed circuit.

## Discussion

Computer-aided model-based methods and tools are being increasingly used in synthetic biology to guide the design of synthetic biochemical pathways so as to achieve user-defined functions and behaviors [9–11].

In this work, in order to obtain a set of guidelines to aid the design of synthetic genetic networks with a predefined functionality (functional modules), we developed a framework using a multi-objective optimization design (MOOD) procedure. Compared to previous studies [25], a novel feature of our framework is that the result of the optimization is already a set of parameters that optimally achieve the desired function and dynamics, as encoded in the objective indexes. Specifying the desired circuit behavior in terms of performance indexes to be optimized has many advantages. The indexes or objectives can be made valid for a whole class of input signals, they may consider other signals in the circuit apart from input and output, the robustness with respect to uncertainty in the circuit parameters can be included, etc. The proper definition of the optimization indexes representing the desired behavior is a key point. An incorrectly specified objective, not properly representing the actual desired behavior, will lead the optimization in a wrong direction, thus returning a parameters set that will give misleading design guidelines. This is a drawback, but easier to handle than setting the thresholds defining the acceptable circuit behavior after a Monte Carlo sampling, for these do not ensure that a solution will be found [25, 27].

The solutions obtained, i.e. the design objectives together with the respective parameter sets, may be clustered hierarchically, or post-processed with any multivariate statistical analysis tool in order to get further insight into the role of the different parameters. The importance of this, is that the spMODE and LD-tools already order the Pareto front solutions with respect to the objective functions. The LD-tool, as a matter of fact, already provides insight into the role of the different solutions. Further statistical processing is very efficient, as only a small set of data has to be processed (the solutions at the Pareto front), and this set is already ordered. This allows us to reveal and understand associations of parameters and functionality. For example, cluster 1 (red) in the Results Section has the highest sensitivity together with the lowest precision. To implement in the wet-lab a system with this functionality, the RBS in gene B has to be weak, and it should be cloned in a low copy plasmid, as reflected by the guidelines obtained for parameters *kp*_*B*_ and *Km*_*B*_*CgB*, respectively. On the contrary, to implement a cluster 2 (blue) system, the guidelines obtained for the same parameters tell us to put gene B also with a weak RBS and but in a high copy plasmid (Fig. 6).

For a given circuit design with a desired functionality, the guidelines for the kinetic parameters (Fig. 6, Table 4) are very useful to decide which biological components to use out of the ones available from a library of biological parts, such as the MIT Registry of Standard Biological Parts [64] by iGEM Foundation, the BIOSS Toolbox [65], or BioFab [66]. In particular, for the I1-FFL, we showed that important tuning knobs are:

- ***Km***_***X***_*CgX*. This is a lumped plasmid copy number and promoter strength, so it can be tuned by selecting the appropriate replication origin of the plasmid and the promoter; for example from the Anderson Promoter library [53>] available at the iGEM Parts Registry.
- *kp*_*X*_ represents the Ribosome Binding Site strength, and is one of the easiest parameters to tune in the wet-lab using, for instance, RBS libraries, the RBS Calculator from Sallis Lab [54], or nucleotides repetition [55].
- ***d***_***X***_ is the protein degradation rate. It also can be tuned globally by changing the growth rate of the microorganism. It also can be tuned by adding a protein degradation tag to include the protein in an active degradation pathway.

As more and more parts are deposited and characterized in these libraries, frameworks providing guidelines for the design and wet-lab implementation, like the ones presented here, will gain more applicability and the design of synthetic genetic circuits will become more rationale-based than intuition-based.

The analysis performed in the Application Scenario I, shows that it is possible to use only one parameter to move from the Pareto front to a sub-optimal solution. For example, starting from a solution with high precision and low sensitivity, one can move to a solution with higher sensitivity and lower precision; with almost no losing optimality. This is very useful in the wet-lab, because it means that once you have the system implemented in the wet-lab, it is possible to change the output of your system in a controlled way by performing the minimum amount of changes to it. The methodology easily allows to check how the initial solution will deteriorate by changing the value of only one parameter (see Fig. 7). Of course, moving along the Pareto front solutions requires modifying more parameters, i.e. changing the values of the parameters from a cluster to another one; however we showed that the obtained guidelines are really robust and that we can use a particular parameter as a tuning knob in the wet-lab implementation.

In the Application Scenario II, we saw that it is straightforward to have an idea of how much the functionality of the system can be compromised by loading it, i.e. by connecting it to another module. The proposed methodology allows to design the system taking this into account. The analysis is computationally efficient, as it has to be performed only for the Pareto front solutions, and not for the whole objective space. Thus, we foresee that extending the approach to the analysis of interconnecting several devices will not be difficult. In a way, as advocated in [5], the approach is less like highly modular electrical engineering, and more like civil and mechanical engineering in its use of optimization of modeling of whole system-level taking into account loads and flows.

Notice that the analysis needs to be performed only along the Pareto front solutions. In this case, we are performing a robustness analysis *a posteriori* with the Pareto optimal solutions approximated. That is, the decision making process is carried out at the end of the MOOD process using additional information, in order to select a *robust* configuration. This is congruent with similar analysis of uncertainties and decision making [67].

If it is required by the decision maker to seek actively for a robust set of solutions, a different approach will be required. That is, in order to get such solutions then the robustness measure analysis should be included *a priori* within the optimisation process. This leads to different optimisation instances known as robust design optimization (RDO) and reliability based design optimisation (RBDO) [68]. The former seeks to minimize the sensitivity of a solution; the latter to provide a measure of risk failure. In any case, such optimization instances are out of the scope of this work and are proposed as future work.

The general applicability of the framework allows to use it with different functional modules and topologies, as soon as the ODEs can be obtained from reactions, although evidently difficulties will arise when dealing with larger networks. In that sense it is interesting to note the difference between the problem of expensive computation and the one of large-scale optimization. Expensive computation arises when the complexity of the system makes the evaluation of the objective function an expensive task. On the contrary, large-scale is related with the amount of decision variables and the size of the objective space. In the cases we are dealing with, this two problems will be more or less coupled. For a larger network, more kinetic parameters (decision variables) and more expensive computation of the dynamics of the system to evaluate the objectives. Nevertheless, one of the key issues will be to obtain a reasonable reduced model of the module to give to the optimization algorithm rather than the optimization itself. Genetic algorithms like spMODE have been used in the past with problems with sizes including 15 objectives and hundreds of decision variables with reasonable computational cost, and related research is a hot topic [69, 70]. Also memory handling in the mentioned algorithms is very efficient, as the only information that propagates from generation to generation is the population.

## Conclusion

The proposed multi-objective optimization design framework is able to provide effective guidelines to tune biological parameters so as to achieve a desired circuit behavior. Moreover, it is easy to analyze the impact of the context on the synthetic device to be designed. That is, one can analyze how the presence of a downstream load influences the performance of the designed circuit, and take it into account. Finally, our results suggest that –although system dynamics actually put constraints on the possible values of the kinetic parameters– design guidelines can be obtained to build a biological systems with a desired functionality.

## Availability of data and materials

All the material used in this work can be found in the following locations:

- The spMODE, a multi-objective optimization algorithm based on differential evolution implemented in MATLAB is available at MatlabCentral, code 39215. http://www.mathworks.com/matlabcentral/fileexchange/39215
- The LD-tool toolbox to help visualization in MATLAB is available at MatlabCentral, code 24042. http://www.mathworks.com/matlabcentral/fileexchange/24042
- An SBML implementation of the I1-FFL model was deposited in BioModels with identifer MODEL1511290000. https://www.ebi.ac.uk/biomodels-main/ (This implementation is not part of the multi-objective optimization design procedure, although it was included for completeness and is intended to be used separately.)
- The source code of the all the software developed for this work is available in the Additional file 2 — matlabscripts.zip and also publicly available at http://sb2cl.ai2.upv.es/content/software, and it is explained in Additional file 1, Section 2. Matlab CODE.

## Endnotes

^1^http://es.mathworks.com/matlabcentral/fileexchange/39215.

^2^ Tool available at http://www.mathworks.com/matlabcentral/fileexchange/24042.

^3^ publicly available at http://sb2cl.ai2.upv.es/content/software.

## Additional files

Additional file 1 additional. (1) I1-FFL model, Tables S1–S2; (2) 2. Matlab CODE (3) Supplementary Tables and Figures: Figure S1 – S4 and Table S3. (PDF 978 kb)

Additional file 2 matlabscripts.zip. Code source of the software explained in Additional file 1 (2. Matlab CODE). (ZIP 255 kb)
