# Supplementary material for: Multi-objective optimization framework to obtain model-based guidelines for tuning biological synthetic devices: an adaptive network case
Source: BMC Syst Biol. 2016 Mar 11;10:27. doi: 10.1186/s12918-016-0269-0 (PMC4788947; doi:10.1186/s12918-016-0269-0)
Supplement: Additional file 1 — additional. (1) I1-FFL model, Tables S1–S2; (2) 2. Matlab CODE (3) Supplementary Tables and Figures: Figure S1 – S4 and Table S3. (PDF 978 kb) [file 12918_2016_269_MOESM1_ESM.pdf]

Additional File 1☆

Yadira Boada[a], Gilberto Reynoso-Meza[b], Jesús Picó[a], Alejandro Vignoni[a,1]

[a]*Institut d'Automàtica i Informàtica Industrial, Universitat Politècnica de València, Valencia, Spain*
[b]*Industrial and Systems Engineering Graduate Program (PPGEPS), Pontifical Catholic University of Parana (PUCPR), Curitiba, Brasil.*

Abstract

In this file there is all the additional information including: Model reduction, explanation of the Matlab code and additional tables and figure to help support the main paper.

1. I1-FFL model

Next, a complete biochemical model of the I1-FFL gene regularoty network is derived, and its corresponding dynamical model based on balance equations and mass action kinetics is formulated. The biochemical reactions considered can be split in two main classes: the *gene expression* reactions, and the *induction* ones. In the *gene expression* block, the main processes considered for each of the three proteins are the binding of the RNA polymerase to the promoter, transcription, translation, mRNA degradation and protein degradation. In the *induction* part, the main processes considered are binding between the protein A and the inducer to form the monomer, monomer degradation, dimer formation and its degradation, addition of external inducer, diffusion of the inducer, inducer degradation, binding of the dimer to the gB promoter, binding of the dimer to the gC promoter, and binding between the activator (or repressor) and the gC hybrid promoter. The corresponding set of variables and corresponding symbols are shown in Table 1.

The resulting set of biochemical reactions is:

☆Boada et al. Obtaining model based guidelines for the design of synthetic devices using a multi-objective optimization framework. An adaptive network case

[1]Actual affiliation is Center for Systems Biology Dresden (CSBD), Max Planck Institute of Molecular Cell Biology and Genetics, Pfotenhauerstr. 108, 01307 Dresden, Germany

Table S 1. List of variables in the complete model.

Variable	Description	Units	Symbol
x_1	DNA promoter gene A	nM	gA
x_2	RNA polymerase	nM	RNAp
x_3	gA·RNAp complex	nM	gA·RNAp
x_4	messenger RNA for gene A	nM	mA
x_5	A protein	nM	A
x_6	Intracellular inducer	nM	I
x_7	A·I monomer	nM	A·I
x_8	$(\text{A·I})_2$ dimer	nM	$(\text{A·I})_2$
x_9	DNA promoter gene B	nM	gB
x_{10}	gB(A·I)_2 complex	nM	gB(A·I)_2
x_{11}	DNA promoter gene C	nM	gC
x_{12}	gC(A·I)_2 complex	nM	gC(A·I)_2
x_{13}	gC·B complex	nM	gC·B
x_{14}	gC·B(A·I)_2 complex	nM	gC·B(A·I)_2
x_{15}	$\text{gB(A·I)}_2\text{RNAp}$ complex	nM	$\text{gB(A·I)}_2\text{RNAp}$
x_{16}	messenger RNA for gene B	nM	mB
x_{17}	B protein	nM	B
x_{18}	messenger RNA for gene C	nM	mC
x_{19}	C protein	nM	C
x_{20}	Extracellular inducer	nM	I_e

gene A:

$$\text{gA} + \text{RNAp} \underset{k_{-1}}{\overset{k_1}{\rightleftharpoons}} \text{gA·RNAp}$$

$$\text{gA·RNAp} \xrightarrow{k_{m_A}} \text{gA} + \text{RNAp} + \text{mA}$$

$$\text{mA} \xrightarrow{k_{p_A}} \text{mA} + \text{A}$$

$$\text{mA} \xrightarrow{d_{m_A}} \emptyset$$

$$\text{A} \xrightarrow{d_A} \emptyset$$

$$\text{A} + \text{I} \underset{k-2}{\overset{k_2}{\rightleftharpoons}} \text{AI}$$

$$\emptyset \xrightarrow{k_e} \text{Ie}$$

$$\text{Ie} \underset{k_d}{\overset{k_d}{\rightleftharpoons}} \text{I}$$

$$\text{I} \xrightarrow{d_I} \emptyset$$

$$2\,(\text{AI}) \underset{k_{-3}}{\overset{k_3}{\rightleftharpoons}} (\text{AI})_2$$

$$(\text{AI}) \xrightarrow{d_{AI}} \emptyset$$

$$(\text{AI})_2 \xrightarrow{d_{AI2}} \emptyset$$

gene B:

$$\mathrm{gB} + (\mathrm{AI})_2 \underset{k_{-4}}{\overset{k_4}{\rightleftharpoons}} \mathrm{gB}{\cdot}(\mathrm{AI})_2$$

$$\mathrm{gB}{\cdot}(\mathrm{AI})_2 + \mathrm{RNAp} \underset{k_{-7}}{\overset{k_7}{\rightleftharpoons}} \mathrm{gB}{\cdot}(\mathrm{AI})_2{\cdot}\mathrm{RNAp}$$

$$\mathrm{gB}{\cdot}(\mathrm{AI})_2{\cdot}\mathrm{RNAp} \xrightarrow{k_{m_B}} \mathrm{gB}{\cdot}(\mathrm{AI})_2 + \mathrm{RNAp} + \mathrm{mB}$$

$$\mathrm{mB} \xrightarrow{k_{p_B}} \mathrm{mB} + \mathrm{B}$$

$$\mathrm{mB} \xrightarrow{d_{m_B}} \emptyset$$

$$\mathrm{B} \xrightarrow{d_B} \emptyset$$

gene C:

$$\mathrm{gC} + (\mathrm{AI})_2 \underset{k_{-5}}{\overset{k_5}{\rightleftharpoons}} \mathrm{gC}{\cdot}(\mathrm{AI})_2$$

$$\mathrm{gC}{\cdot}\mathrm{B} + (\mathrm{AI})_2 \underset{k_{-6}}{\overset{k_6}{\rightleftharpoons}} \mathrm{gC}{\cdot}\mathrm{B}{\cdot}(\mathrm{AI})_2$$

$$\mathrm{gC}{\cdot}(\mathrm{AI})_2 + \mathrm{B} \underset{k_{-8}}{\overset{k_8}{\rightleftharpoons}} \mathrm{gC}{\cdot}\mathrm{B}{\cdot}(\mathrm{AI})_2$$

$$\mathrm{gC} + \mathrm{B} \underset{k_{-9}}{\overset{k_9}{\rightleftharpoons}} \mathrm{gC}{\cdot}\mathrm{B}$$

$$\mathrm{gC}{\cdot}(\mathrm{AI})_2 + \mathrm{RNAp} \underset{k_{-10}}{\overset{k_{10}}{\rightleftharpoons}} \mathrm{gC}{\cdot}(\mathrm{AI})_2{\cdot}\mathrm{RNAp}$$

$$\mathrm{gC}{\cdot}(\mathrm{AI})_2{\cdot}\mathrm{RNAp} \xrightarrow{k_{m_C}} \mathrm{gC}{\cdot}(\mathrm{AI})_2 + \mathrm{RNAp} + \mathrm{mC}$$

$$\mathrm{mC} \xrightarrow{k_{p_C}} \mathrm{mC} + \mathrm{C}$$

$$\mathrm{mC} \xrightarrow{d_{m_C}} \emptyset$$

$$\mathrm{C} \xrightarrow{d_C} \emptyset$$

Note: the empty set $\emptyset$ denotes species degradation when placed in the right hand side of a reaction.

Notice we assume that binding between the activating dimer and the gC hybrid promoter is always possible, even if the repressor B is already bound to the promoter, and vice versa. Yet, we consider that whenever the repressor B is bound to the promoter gC, the RNA polymerase cannot bind the promoter.

Using the law of mass-action kinetics (Horn and Jackson, 1972; Chellaboina et al., 2009), the previous reactions can be used to formulate the corresponding dynamic balances of the species

concentrations:

$$\dot{x_1} = -k_1x_1x_2 + k_{-1}x_3 + k_{mA}x_3$$
$$\dot{x_2} = -k_1x_1x_2 + k_{-1}x_3 + k_{mA}x_3 - k_7x_{10}x_2 + k_{-7}x_{15} + k_{mB}x_{15}$$
$$\dot{x_3} = k_1x_1x_2 - k_{-1}x_3 - k_{mA}x_3$$
$$\dot{x_4} = k_{mA}x_3 - d_{mA}x_4$$
$$\dot{x_5} = k_{pA}x_4 - d_Ax_5 - k_2x_5x_6 + k_{-2}x_7$$
$$\dot{x_6} = -k_2x_5x_6 + k_{-2}x_7 + k_dx_{20} - k_dx_6 - d_Ix_6$$
$$\dot{x_7} = k_2x_5x_6 - k_{-2}x_7 - 2k_3x_7^2 + 2k_{-3}x_8 - d_{AI}x_7$$
$$\dot{x_8} = k_3x_7^2 - k_{-3}x_8 - k_4x_8x_9 + k_{-4}x_{10} - k_5x_8x_{11} + k_{-5}x_{12} - k_6x_8x_{13} + k_{-6}x_{14} - d_{AI2}x_8$$
$$\dot{x_9} = -k_4x_9x_8 + k_{-4}x_{10}$$
$$\dot{x_{10}} = k_4x_9x_8 - k_{-4}x_{10} - k_7x_{10}x_2 + k_{-7}x_{15} + k_{mB}x_{15}$$
$$\dot{x_{11}} = -k_9x_{11}x_{17} + k_{-9}x_{13} - k_5x_{11}x_8 + k_{-5}x_{12}$$
$$\dot{x_{12}} = k_5x_{11}x_8 - k_{-5}x_{12} - k_8x_{12}x_{17} + k_{-8}x_{14}$$
$$\dot{x_{13}} = k_9x_{11}x_{17} - k_{-9}x_{13} - k_6x_{13}x_8 + k_{-6}x_{14}$$
$$\dot{x_{14}} = k_6x_{13}x_8 - k_{-6}x_{14} + k_8x_{12}x_{17} - k_{-8}x_{14}$$
$$\dot{x_{15}} = k_7x_{10}x_2 - k_{-7}x_{15} - k_{mB}x_{15}$$
$$\dot{x_{16}} = k_{mB}x_{15} - d_{mB}x_{16}$$
$$\dot{x_{17}} = k_{pB}x_{16} - d_Bx_{17} - k_9x_{11}x_{17} + k_{-9}x_{13} - k_8x_{12}x_{17} + k_{-8}x_{14}$$
$$\dot{x_{18}} = k_{mC}x_{12} - d_{mC}x_{18}$$
$$\dot{x_{19}} = k_{pC}x_{18} - d_Cx_{19}$$
$$\dot{x_{20}} = k_dx_6 - k_dx_{20} - d_Ix_{20}$$

Note these equations can be derived either by inspection, or using specific software to automate the process. Software packages like BioNetGen (Blinov et al., 2004) or COPASI (Mendes et al., 2009) allow to obtain the dynamic kinetic model from either the set of reactions or from SBML files encoding them.

The complete model is of large order, which implies a high computational cost for the parameters estimation process that will be carried out later on. Moreover, the large differences in the time scales among the different species in the synthetic gene network (typically many orders of magnitude) originate huge difficulties for simulating the temporal evolution of the network and for understanding the basic principles of its operation. Therefore, the dynamical model will be reduced using time-scale separation and detection of invariant moieties.

We apply the *Quasi Steady-State Approximation* (QSSA) to the fast chemical species (Zagaris et al., 2004; Mélykúti et al., 2014). In essence QSSA is a singular perturbation method that considers the time-scale separation among the different dynamics. In particular, we will assume that binding reactions occur very fast in comparison with those corresponding to transcription, translation or even genuine degradation. On the other hand, monomer formation is faster that dimerization Therefore, the differential equation corresponding to the AI complex formation will be assumed to be at steady state.

Additional algebraic relationships among variables can be obtained through *system invariants*. In the case of reaction networks, it can be observed that some reactions are a linear combi-

Table S 2. List of variables used in the reduced model.

Variable	Description	Units	Symbol
x_1	$\mathrm{mRNA_{gA}}$	nM	mA
x_2	A protein	nM	A
x_3	Inducer	nM	I
M	A·I monomer	nM	A·I
x_4	$(\mathrm{A{\cdot}I})_2$ dimer	nM	$(\mathrm{A{\cdot}I})_2$
x_5	$\mathrm{mRNA_{gB}}$	nM	mB
x_6	B protein	nM	B
x_7	$\mathrm{mRNA_{gC}}$	nM	mC
x_8	C protein	nM	C
x_9	External $\mathrm{I_{ext}}$	nM	I_e

nation of other ones. Then, the linear combination of the concentrations of the species involved will keep constant in time. These linear combinations, so called moieties, can be understood as a kind of quasi-species that keep invariant, i.e. keep constant concentration.

After the reduction, ommited here, we get a system of nine ordinary differential equations (each one corresponding to the dynamics of one of the species) plus an algebraic equation, and 26 model parameters that will be the decision variables at the the optimization step. The resulting model is:

$$\dot{x}_1 = k_{mA} C_{gA} - d_{mA} x_1 \quad (1)$$

$$\dot{x}_2 = k_{pA} x_1 - d_A x_2 - k_2 x_2 x_3 + k_{-2} M \quad (2)$$

$$\dot{x}_3 = -k_2 x_2 x_3 + k_{-2} M + k_d x_9 - k_d x_3 - d_I x_3 \quad (3)$$

$$\dot{x}_4 = k_3 M^2 - 2k_{-3} x_4 - d_{AI2} x_4 \quad (4)$$

$$\dot{x}_5 = K_{mB}\, C_{gB} \frac{x_4}{\theta_1 + x_4} - d_{mB} x_5 \quad (5)$$

$$\dot{x}_6 = k_{pB} x_5 - d_B x_6 \quad (6)$$

$$\dot{x}_7 = K_{mC} C_{gC} \frac{x_4 + \beta_1 x_6 + \beta_2 x_4 x_6}{\theta_2 + \theta_3 x_4 + \theta_4 x_6 + \theta_5 x_4 x_6} - d_{mC} x_7 \quad (7)$$

$$\dot{x}_8 = k_{pC} x_7 - d_C x_8 \quad (8)$$

$$\dot{x}_9 = -k_d x_9 + k_d x_3 - d_{Ie} x_9 \quad (9)$$

with $M = -\frac{d_{AI}+k_{-2}}{2k_3} + \frac{1}{2k_3}\sqrt{(d_{AI} + k_{-2})^2 + 4k_3(k_2 x_2 x_3 + 2k_{-3} x_4)}$.

The resulting set of biochemical reactions corresponding to the reduced model is:

gene A:

$$\emptyset \xrightarrow{f_A} +\text{mA}$$

$$\text{mA} \xrightarrow{k_{p_A}} \text{mA} + \text{A}$$

$$\text{mA} \xrightarrow{d_{m_A}} \emptyset$$

$$\text{A} \xrightarrow{d_A} \emptyset$$

$$2\text{A} + 2\text{I} \underset{2k-3}{\overset{k_2 M}{\rightleftharpoons}} (\text{AI})_2$$

$$\emptyset \xrightarrow{k_e} \text{Ie}$$

$$\text{Ie} \underset{k_d}{\overset{k_d}{\rightleftharpoons}} \text{I}$$

$$\text{I} \xrightarrow{d_I} \emptyset$$

$$(\text{AI})_2 \xrightarrow{d_{AI2}} \emptyset$$

gene B:

$$\emptyset \xrightarrow{f_B} \text{mB}$$

$$\text{mB} \xrightarrow{k_{p_B}} \text{mB} + \text{B}$$

$$\text{mB} \xrightarrow{d_{m_B}} \emptyset$$

$$\text{B} \xrightarrow{d_B} \emptyset$$

gene C:

$$\emptyset \xrightarrow{f_C} +\text{mC}$$

$$\text{mC} \xrightarrow{k_{p_C}} \text{mC} + \text{C}$$

$$\text{mC} \xrightarrow{d_{m_C}} \emptyset$$

$$\text{C} \xrightarrow{d_C} \emptyset$$

Where f_A, f_B, and f_C are the lumped propensities obtained from the reduction:

$$f_A = k_{mA} C_{gA}, \tag{10}$$

$$f_B = K_{mB}\, C_{gC} \frac{x_4}{\theta_1 + x_4}, \tag{11}$$

$$f_C = K_{mC} C_{gC} \frac{x_4 + \beta_1 x_6 + \beta_2 x_4 x_6}{\theta_2 + \theta_3 x_4 + \theta_4 x_6 + \theta_5 x_4 x_6}. \tag{12}$$

2. Matlab CODE

A short description of the main functions integrating this code and justification of the value sets is given below. It has been divided in two groups: files related to the model computational characterization, and files used by the optimizer, which link to the first set.

Model code

- ***model_3genes.m*** is a function for the ODEs of the reduced model. Receives the value of the state vector x at time t, the parameters, initial conditions and time point; and returns a vector with the derivatives defined in it. When used with the command `ode23s` in the function *objective_func.m* one obtains the solution of the ODEs system for the given parameters.

- ***objective_func.m*** is the objective function. It receives the parameters and returns the **objectives values vector**, after calculating J_1 and J_2 for the corresponding dynamic response obtained with the given parameters.

  The 14 variables are initialized, and the 10 parameters are not because the optimizer will work with a given range in its code.

  The *ode23s* algorithm gives the variables values Y for each t, using *model_3genes.m*. This *ode* algorithm was selected because our system model is what it is known as *stiff*, in terms of the numerical solution of ordinary differential equations, i.e. it has both slow and fast dynamics. An ordinary differential equation problem is stiff if the solution being sought is varying slowly, but there are nearby solutions that vary rapidly, so the numerical method must take small steps to obtain satisfactory results. For our integration problem we use an absolute tolerance of 1e-8, and a relative tolerance of 1e-6.

  For computational simulation, we start from the equilibrium initial conditions (precomputed) and give a jump of 50nM to the concentration of x_9 to simulate the induction. With respect to the simulation parameters, the simulation sampling time (Ts) was fixed to $1e^{-3}$ minutes, and a total simulation time $Tsim = 300$ minutes was used.

- ***eval_obj_fun.m*** is the function that receive a population of parameters, evaluates the objective functions in this population, and accumulates the results in a matrix to return it. It is executed at each iteration of the sp-MODE algorithm.

MOO code

First, highlight that we use the script ***Tutorial.m*** to run all the functions used to obtain the results shown in the main paper.

The first step is to run the *spMODEparam* file to build the variable 'spMODEDat' with the variables required for the optimization. Here the number of objectives are defined, also the number of decision variables and the *'Cost Function'*, which brings the objectives matrix after previous *ode* simulations (by means of interlinked functions mentioned above, constituting in essence the problem 'nucleus' or characterization). The field of search, and bounds to improve pertinency of solutions in the objective space so as to cut solutions with no interest to the DM, are defined here too. Also other aspects, such as maximum Pareto optimal solutions required and a bound on the number of function evaluations.

Once the Pareto set and the Pareto front are found by the optimizer, results can be plot with optional features through the *Leveltool*. This tool provides the LD visualization for the MCDM.

- ***spMODEparam.m*** generates the required parameters to run the spMODE optimization algorithm.

  In this file the variables regarding the multi-objective problem are defined. The values of interest for our problem are:

  1. Number of objectives.
     spMODEDat.NOBJ = 2
  2. Number of decision variables.
     spMODEDat.NVAR = 10
  3. Cost Function.
     spMODEDat.mop = str2func('CostFunction')
  4. Problem Instance.
     spMODEDat.CostProblem = 'modelo3genes'
  5. Maximum and minimum values for the parameters or decision variables are fixed in order to give a range to the optimizer to search the optimal solutions, (spMODEDat.FieldD). k_d and d_{I_e} were fixed to avoid the optimizer to modify the model input I_e, as we want an step input determined by $K_e(t)$.
  6. Bounds on objectives.
     spMODEDat.Pertinency=[1e-3 200; 1e-4 20]; A row for each objective, with the minimum and maximum values desired.

- ***CostFunction.m*** calls the cost function of your own multi-objective problem. In this case *eval_obj_fun.m*. It also includes a default mechanism to improve pertinency (Objective space bounded).

Clustering and Visualization

- ***clustering.m*** is a script that performs the hierarchical clustering with the solutions obtained from the spMODE optimization algorithm, and uses the modified LD-tool to plot the LD plots with the cluster number as Y-axis.

Computational cost. Execution of our MOO using the sp-MODE algorithm (15.000 evaluations of the objective function) took around 10 hours and 25 minutes and was performed in a Intel XEON Server with 12 cores and 32 Gb of RAM Memory.

3. Supplementary Tables and Figures

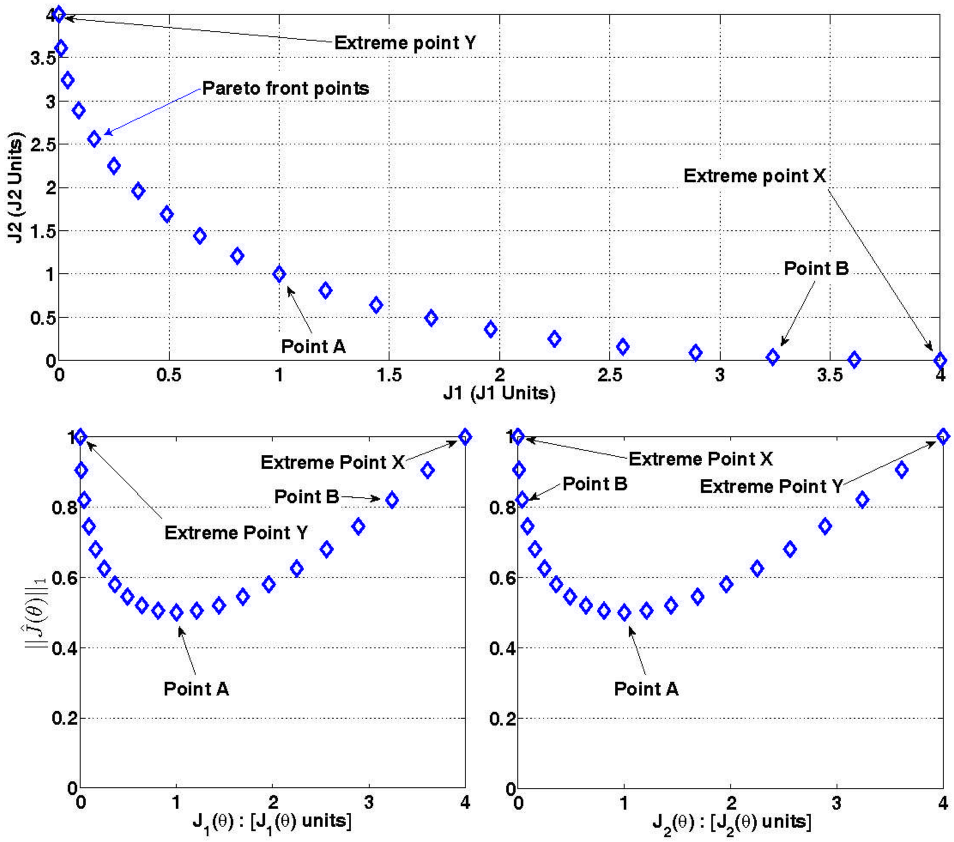

Figure S 1. Example of Level Diagram for a bi-objective Pareto front and set.

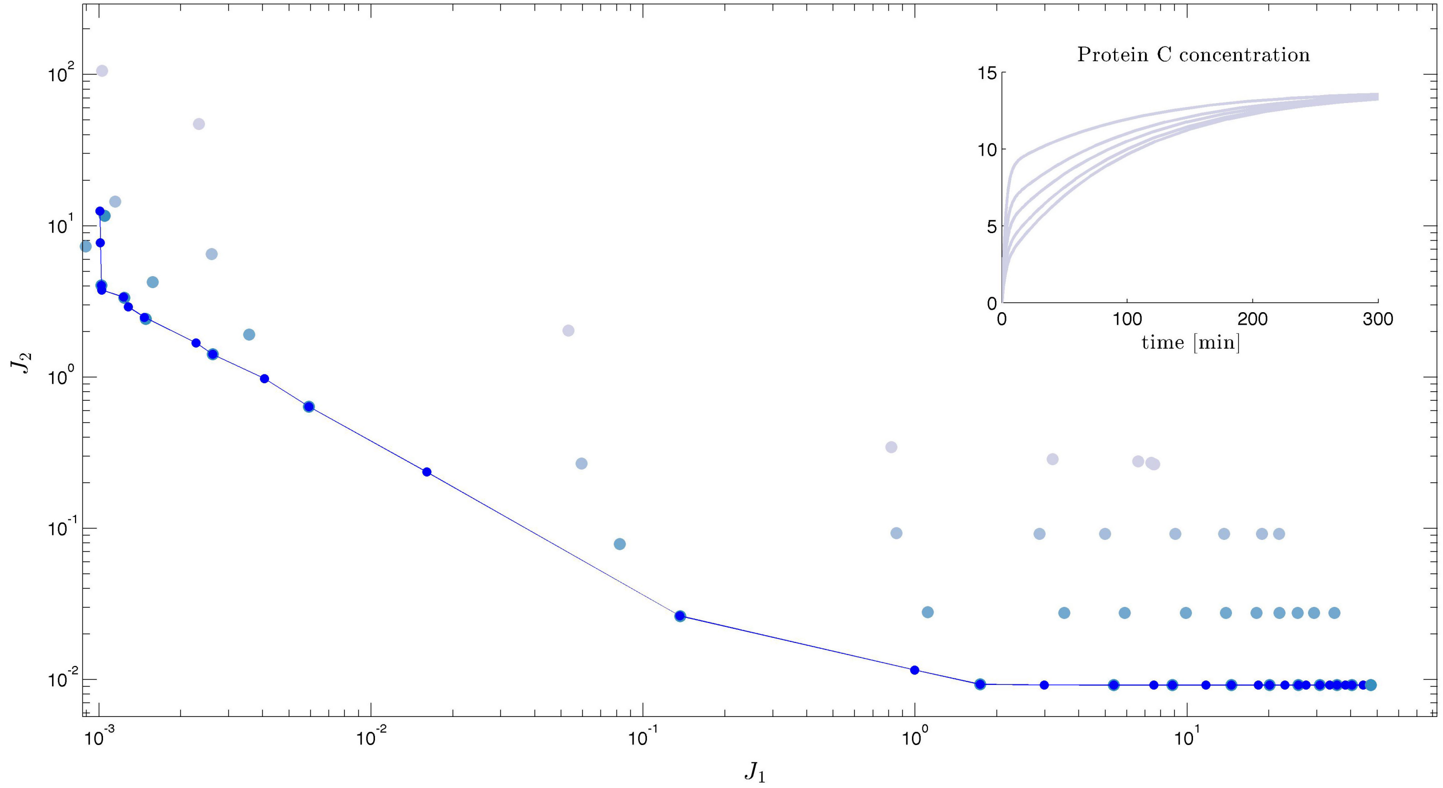

Figure S 2. Pareto Front in blue line connected dots. Dots changing from blue to light blue are obtained by changing the degradation rate of protein C represented by $d_C \in [0.3\ 0.1\ 0.03\ 0.01]$ starting at the extreme solution. Notice, that decreasing d_C lead to a complete lose of optimality and moreover of the adaptation behavior, as we can see in the temporal profile of the protein C concentration in the inset.

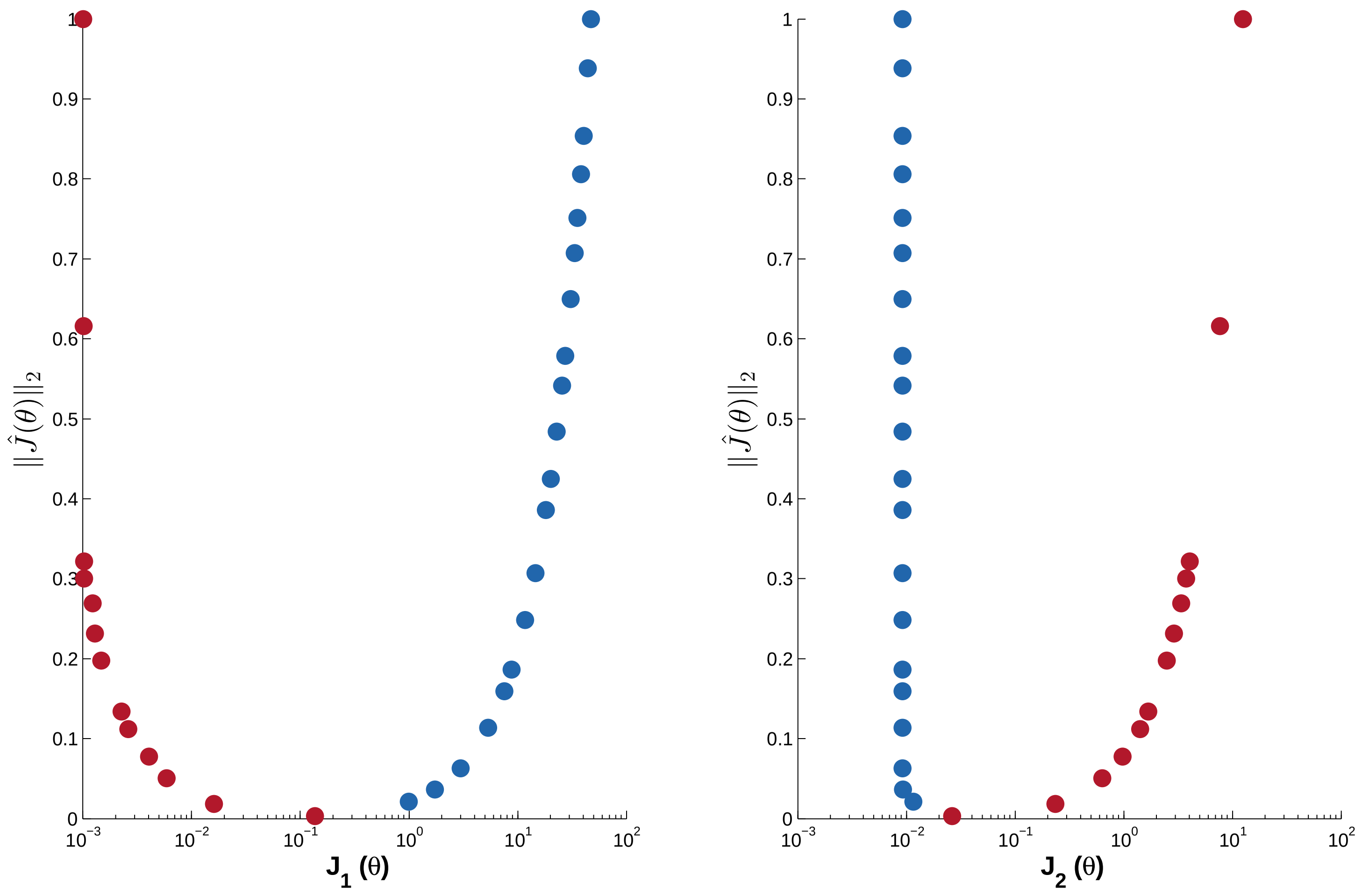

Figure S 3. Pareto Front as the distance to ideal point. $J_1(\theta)$ is the sensitivity and $J_2(\theta)$ is the precision objectives. Cluster 1 is plotted in red circles and cluster 2 is plotted in blue circles.

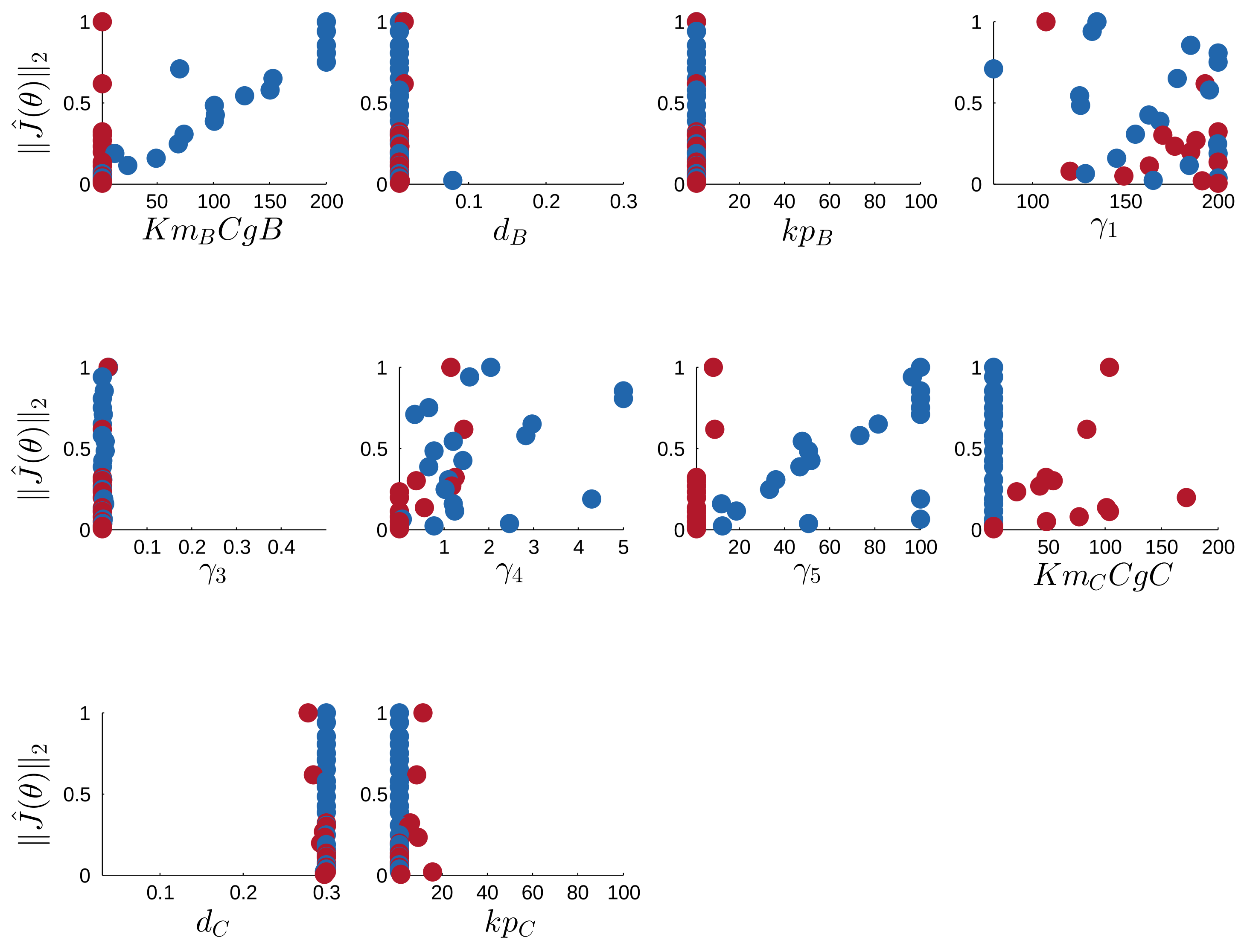

Figure S 4. Pareto set of decision variables. Each parameter of the model was plotted according its distance to ideal point. Red circles represent the cluster 1 and blue circles allow to the cluster 2.

Table S 3. Solutions obtained from the MOO. Columns are values for the design objectives and parameters. Rows are the solutions obtained.

**Solution**	J_1	J_2	Km_CCgC	Km_BCgB	d_B	d_C	γ_1	γ_3	γ_4	γ_5	kp_B	kp_C
**1**	0.00	12.53	104.09	1.00	0.02	0.28	107.41	0.01	1.15	8.56	1.00	11.43
**2**	0.00	7.72	84.06	1.00	0.02	0.28	193.06	0.00	1.44	9.25	1.00	8.66
**3**	0.00	4.04	47.71	1.00	0.01	0.30	200.00	0.00	1.25	1.00	1.00	5.93
**4**	0.00	3.76	53.93	1.00	0.01	0.30	170.42	0.00	0.38	1.00	1.00	5.08
**5**	0.00	3.38	41.92	1.00	0.01	0.30	188.31	0.00	1.17	1.00	1.00	5.68
**6**	0.00	2.91	21.52	1.00	0.01	0.30	176.89	0.00	0.00	1.00	1.00	9.44
**7**	0.00	2.48	171.91	1.00	0.01	0.29	185.33	0.00	0.00	1.00	1.00	1.00
**8**	0.00	1.68	101.48	1.00	0.01	0.30	200.00	0.00	0.57	1.00	1.00	1.15
**9**	0.00	1.41	103.74	1.00	0.01	0.30	163.00	0.00	0.00	1.00	1.00	1.00
**10**	0.00	0.98	77.26	1.00	0.01	0.30	120.21	0.00	0.00	1.00	1.00	1.00
**11**	0.01	0.64	47.90	1.00	0.01	0.30	149.24	0.00	0.00	1.00	1.00	1.00
**12**	0.02	0.24	1.00	1.00	0.01	0.30	191.70	0.00	0.00	1.00	1.00	15.68
**13**	0.14	0.03	1.00	1.00	0.01	0.30	200.00	0.00	0.00	1.00	1.00	1.80
**14**	0.99	0.01	1.00	1.17	0.08	0.30	165.00	0.00	0.78	12.66	1.00	1.00
**15**	1.73	0.01	1.00	1.00	0.01	0.30	200.00	0.00	2.47	50.50	1.00	1.00
**16**	2.98	0.01	1.00	1.00	0.01	0.30	128.61	0.00	0.07	100.00	1.00	1.00
**17**	5.37	0.01	1.00	24.05	0.01	0.30	184.64	0.00	1.24	18.72	1.00	1.00
**18**	7.54	0.01	1.00	49.25	0.01	0.30	145.35	0.01	1.21	12.13	1.00	1.00
**19**	8.82	0.01	1.00	12.53	0.01	0.30	200.00	0.00	4.30	100.00	1.00	1.00
**12**	11.73	0.01	1.00	68.79	0.01	0.30	199.76	0.00	1.02	33.42	1.00	1.00
**21**	14.52	0.01	1.00	73.80	0.01	0.30	155.42	0.00	1.10	36.26	1.00	1.00
**22**	18.25	0.01	1.00	100.50	0.01	0.30	168.85	0.00	0.66	46.72	1.00	1.00
**23**	20.10	0.01	1.00	101.56	0.01	0.30	162.82	0.00	1.42	51.65	1.00	1.00
**24**	22.90	0.01	1.00	100.50	0.01	0.30	126.03	0.01	0.77	50.50	1.00	1.00
**25**	25.61	0.01	1.00	127.78	0.01	0.30	125.51	0.01	1.21	47.80	1.00	1.00
**26**	27.38	0.01	1.00	150.25	0.01	0.30	195.38	0.00	2.83	73.36	1.00	1.00
**27**	30.74	0.01	1.00	152.69	0.01	0.30	178.20	0.00	2.97	81.41	1.00	1.00
**28**	33.45	0.01	1.00	70.15	0.01	0.30	78.93	0.00	0.35	100.00	1.00	1.00
**29**	35.51	0.01	1.00	200.00	0.01	0.30	200.00	0.00	0.66	100.00	1.00	1.00
**30**	38.11	0.01	1.00	200.00	0.01	0.30	200.00	0.00	5.00	100.00	1.00	1.00
**31**	40.38	0.01	1.00	200.00	0.01	0.30	185.33	0.00	5.00	100.00	1.00	1.00
**32**	44.39	0.01	1.00	200.00	0.01	0.30	132.08	0.00	1.57	96.57	1.00	1.00
**33**	47.29	0.01	1.00	200.00	0.01	0.30	134.69	0.01	2.05	100.00	1.00	1.00

4. References

Blinov, M. L., Faeder, J. R., Goldstein, B., Hlavacek, W. S., 2004. Bionetgen: software for rule-based modeling of signal transduction based on the interactions of molecular domains. Bioinformatics 20 (17), 3289–3291.

Chellaboina, V., Bhat, S., Haddad, M., Bernstein, D. S., 2009. Modeling and analysis of mass-action kinetics. Control Systems, IEEE 29 (4), 60–78.

Horn, F., Jackson, R., 1972. General mass action kinetics. Archive for rational mechanics and analysis 47 (2), 81–116.

Mélykúti, B., Hespanha, J. P., Khammash, M., 2014. Equilibrium distributions of simple biochemical reaction systems for time-scale separation in stochastic reaction networks. Journal of The Royal Society Interface 11 (97), 20140054.

Mendes, P., Hoops, S., Sahle, S., Gauges, R., Dada, J., Kummer, U., 2009. Computational modeling of biochemical networks using copasi. In: Systems Biology. Springer, pp. 17–59.

Zagaris, A., Kaper, H. G., Kaper, T. J., 2004. Analysis of the computational singular perturbation reduction method for chemical kinetics. Journal of Nonlinear Science 14 (1), 59–91.
